# Murine CAR19 Tregs suppress acute graft-versus-host disease and maintain graft-versus-tumor responses

**DOI:** 10.1172/jci.insight.160674

**Published:** 2022-09-08

**Authors:** Sara Bolivar-Wagers, Michael L. Loschi, Sujeong Jin, Govindarajan Thangavelu, Jemma H. Larson, Cameron S. McDonald-Hyman, Ethan G. Aguilar, Asim Saha, Brent H. Koehn, Mehrdad Hefazi, Mark J. Osborn, Michael C. Jensen, John E. Wagner, Christopher A. Pennell, Bruce R. Blazar

**Affiliations:** 1 Department of Pediatrics, Division of Pediatric Blood and Marrow Transplantation & Cellular Therapy, and; 2 Department of Medicine, Division of Hematology, Oncology and Transplantation, University of Minnesota Medical School, Minneapolis, Minnesota, USA.; 3 Department of Internal Medicine, Division of Hematology, Mayo Clinic, Rochester, Minnesota, USA.; 4 Department of Pediatrics, Division of Hematology and Oncology, University of Washington, Seattle, Washington, USA.; 5 Department of Laboratory Medicine and Pathology, University of Minnesota Medical School, Minneapolis, Minnesota, USA.

**Keywords:** Immunology, Transplantation, Bone marrow transplantation, Cancer immunotherapy, Stem cell transplantation

## Abstract

Allogeneic hematopoietic stem cell transplantation (allo-HSCT) efficacy is complicated by graft-versus-host disease (GVHD), a leading cause of morbidity and mortality. Regulatory T cells (Tregs) have shown efficacy in preventing GVHD. However, high Treg doses are often required, necessitating substantial ex vivo or in vivo expansion that may diminish suppressor function. To enhance in vivo suppressor function, murine Tregs were transduced to express an anti–human CD19 chimeric antigen receptor (hCAR19) and infused into lethally irradiated, hCD19-transgenic recipients for allo-HSCT. Compared with recipients receiving control transduced Tregs, those receiving hCAR19 Tregs had a marked decrease in acute GVHD lethality. Recipient hCD19 B cells and murine hCD19 TBL12-luciferase (TBL12^luc^) lymphoma cells were both cleared by allogeneic hCAR19 Tregs, which was indicative of graft-versus-tumor (GVT) maintenance and potentiation. Mechanistically, hCAR19 Tregs killed syngeneic hCD19^+^ but not hCD19^–^ murine TBL12^luc^ cells in vitro in a perforin-dependent, granzyme B–independent manner. Importantly, cyclophosphamide-treated, hCD19-transgenic mice given hCAR19 cytotoxic T lymphocytes without allo-HSCT experienced rapid lethality due to systemic toxicity that has been associated with proinflammatory cytokine release; in contrast, hCAR19 Treg suppressor function enabled avoidance of this severe complication. In conclusion, hCAR19 Tregs are a potentially novel and effective strategy to suppress GVHD without loss of GVT responses.

## Graphical abstract

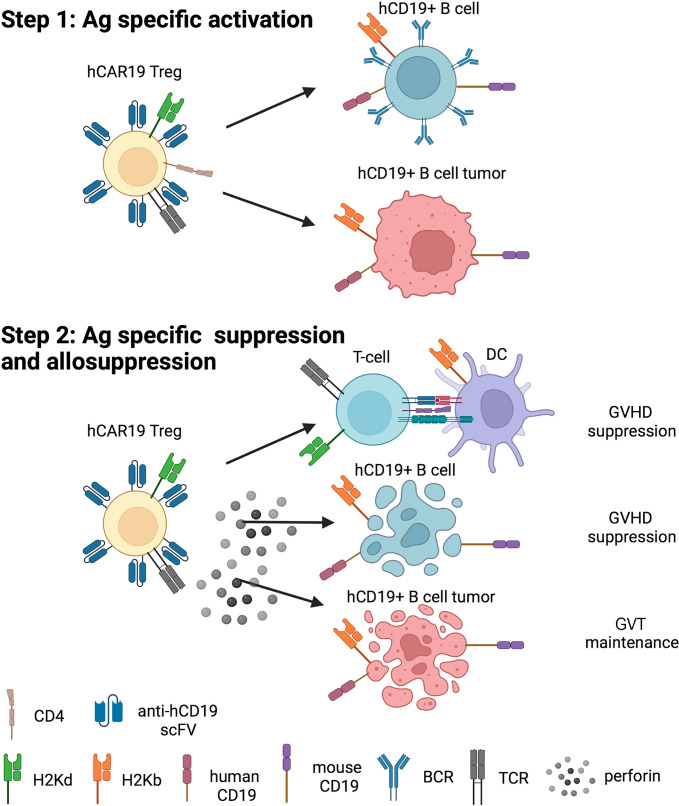

## Introduction

Allogeneic hematopoietic stem cell transplantation (allo-HSCT) can be a curative therapy for hematological malignancies (1, 2). However, a leading consequence of allo-HSCT is graft-versus-host disease (GVHD), an immune-mediated multiorgan inflammatory disease and a leading cause of morbidity and mortality after allo-HSCT (3). Despite current GVHD prophylactic regimens, 30% to 70% of patients with allo-HSCT still develop GVHD, leaving patients more susceptible to infection and relapse (3–8). Preclinical and clinical studies show that adoptive transfer of regulatory T cells (Tregs) can be highly effective at preventing GVHD (9–16). However, clinical translation has been hampered by the requirement for high Treg doses and variability in Treg potency to achieve therapeutic effects.

On a per-cell basis, antigen-specific Tregs are superior to polyclonal Tregs as suppressors of cognate antigen responses. By restricting antigen specificity, the risk for systemic immune suppression may be diminished and the effective cell dose reduced (17). Chimeric antigen receptors (CARs) can redirect Tregs to a desired antigen (18). For example, anti–HLA-A2–specific CAR Tregs suppress HLA-A2 disparate solid organ graft rejection (17). Although this approach targets one of the most common HLA class I allelic mismatched antigens, HLA-A2 is present in only 34.6% of African Americans (19). Thus, there is a need to redirect Tregs toward more readily available target antigens. CAR T cells also are being investigated for the treatment of autoimmunity. For example, CD19-directed CAR (CAR19) T cells can effectively treat murine systemic lupus erythematous by killing B cells (20), and T cells expressing chimeric autoantigen receptors can engage in targeted killing of autoreactive B cells (21).

Tregs use a variety of pathways to mediate suppression, including directed cytolytic activity (22–29). Murine Tregs can regulate immune responses via killing of antigen-presenting B cells in a granzyme B–dependent (GzB-dependent), partially perforin-dependent manner (23). Additionally, human Tregs redirected through bispecific T cell engagers maintain suppression while killing antigen-expressing tumor cells in a perforin-dependent, partially GzB-dependent manner (30). Although MacDonald et al. in 2016 reported human HLA-A2–specific CAR Treg killing of HLA-A2^+^ targets in vitro (17), to date only Boroughs et al. have reported in vitro and in vivo killing by human CAR19 Tregs (31). This group demonstrated that human CAR19 Tregs killed CD19^+^ targets in vitro via the perforin/granzyme pathway, with measurable but low killing of antigen-expressing targets in vivo, using a skin allograft model (31). CAR Tregs could theoretically engage in targeted killing while simultaneously performing immunosuppression.

Activation of conventional CAR19 T cells by CD19^+^ targets triggers the release of proinflammatory cytokines (e.g., TNF-α, IFN-γ), which, in turn, induce endogenous myeloid and endothelial cells to secrete additional proinflammatory cytokines (e.g., IL-1β, IL-6). These amplifying waves of inflammation cause toxicities, such as cytokine release syndrome (CRS), an acute systemic inflammatory response with fever and multiorgan dysfunction, and immune effector cell–associated neurotoxicity syndrome (32–34). Because CAR19 Tregs can kill CD19^+^ targets directly, we reasoned CAR19 Tregs would have antitumor efficacy in vivo. Because Tregs also blunt immune activation of bystander cells, we hypothesized that CAR19 Tregs would be superior to conventional CAR19 T cells by reducing toxicities caused by systemic inflammation. To test our hypothesis, we developed a mouse model that replicated allo-HSCT for CD19^+^ B cell malignancies.

We employed CAR technology to redirect murine Tregs toward human CD19–expressing (hCD19-expressing) B cells. B cells are an ideal target in allo-HSCT models based on their tissue distribution, capacity for antigen presentation, and activation of alloreactive T cells (35), as well as expression on leukemias and lymphomas. We used a murine syngeneic model in which the hCD19 transgene is expressed at hemizygous levels in recipient murine B cells. We employed hemizygotes because homozygous hCD19 expression reduces absolute B cell numbers, and hence the overall number of hCD19 B cells and the mean hCD19 density on B cells exceeds that of B cell lymphoid malignancies as well as nonmalignant B cells (36). Here, we investigated the potential of murine anti–hCD19 CAR (hCAR19) Tregs to deplete recipient hCD19^+^ B cells in vivo and induce systemic toxicity compared with CAR19 T cells. We then evaluated whether hCAR19 Tregs could eliminate murine B cell lymphoma cells expressing hCD19 (hCD19^+^ TBL12) cells in vivo without toxicity. Last, we tested the potential of hCAR19 Tregs to suppress acute GVHD (aGVHD) without abrogating the graft-versus-tumor (GVT) response in a murine major histocompatibility complex (MHC) mismatch allo-HSCT model.

## Results

### Generation of hCAR19 Tregs using retroviral vectors.

**Figure 1:**
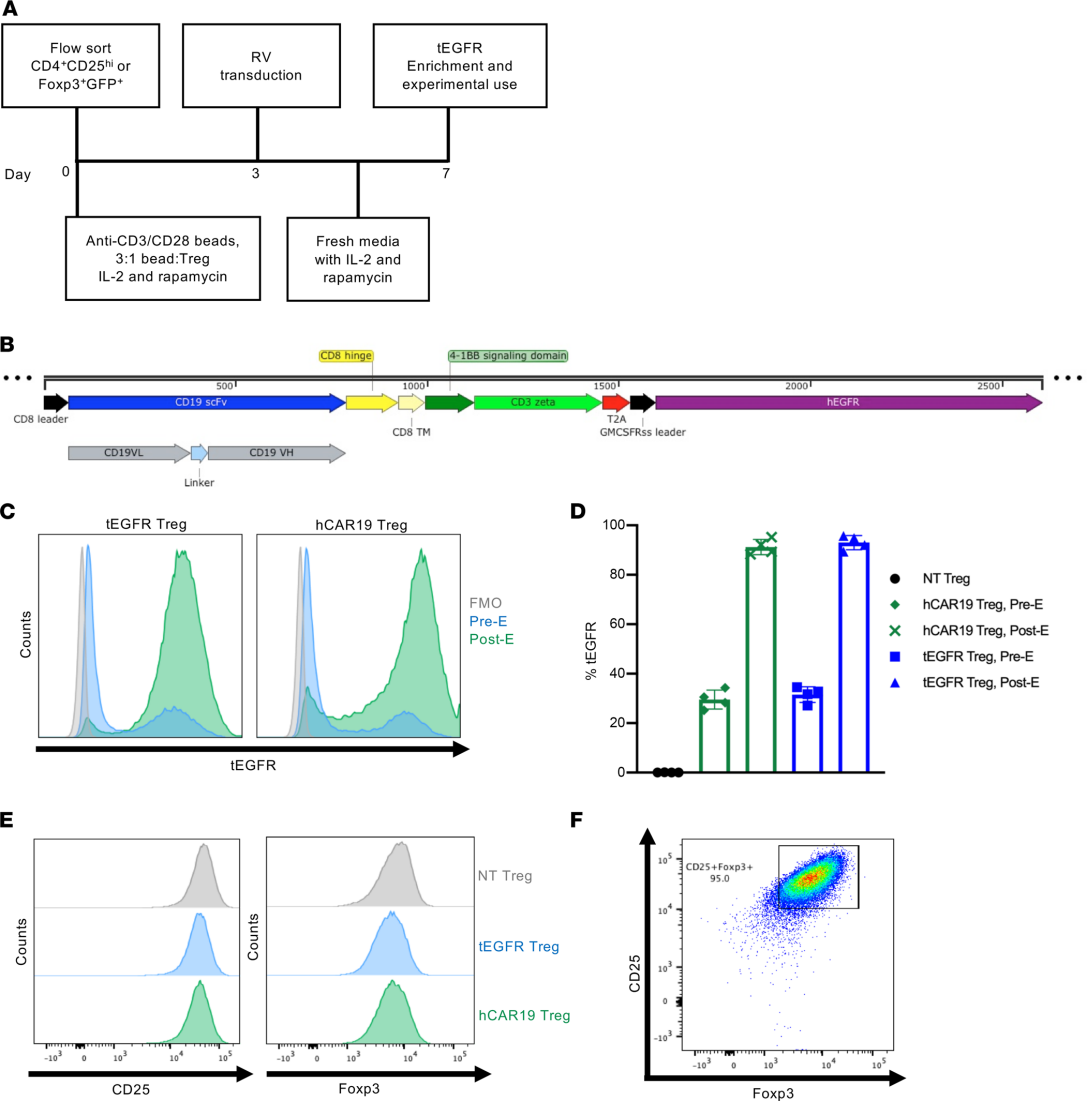
Generation of hCAR19 Tregs. (**A**) Schema of hCAR19 and tEGFR Treg generation. (**B**) Schematic representation of hCAR19 construct in a pMP71 vector backbone. scFv, single chain variable fragment; VL, variable light chain; VH, variable heavy chain; TM, transmembrane domain; GMCSFRss, GM-CSF receptor α chain signal sequences. (**C**) Representative histogram plots of truncated human epidermal growth factor receptor (tEGFR) expression in hCAR19 and tEGFR control transduced Tregs prior to experimental use. FMO, fluorescence minus one; pre-E, before tEGFR-positive enrichment; post-E, following tEGFR-positive enrichment. (**D**) Percentage of tEGFR expression in nontransduced (NT) Tregs, before and after tEGFR enrichment of hCAR19 and tEGFR transduced Tregs. (**E**) Histogram plots of CD25 and Foxp3 expression in NT, tEGFR, and hCAR19 Tregs. (**F**) Representative FACS plot of CD25^+^Foxp3^+^ transduced hCAR19 Tregs prior to experimental use.

Tregs were first enriched from WT or Foxp3-GFP^+^ mice by magnetic-activated cell sorting (MACS) and then flow-sorted for CD4^+^CD25^hi^ or CD4^+^CD25^hi^GFP^+^ cells (Supplemental Figure 1; supplemental material available online with this article; https://doi.org/10.1172/jci.insight.160674DS1). Tregs were activated for 3 days with anti-CD3/CD28 Dynabeads and human IL-2 prior to retroviral transduction (Figure 1A). hCAR19 Tregs were generated using a pMP71 retroviral vector encoding a single chain variable fragment derived from an hCD19-specific monoclonal antibody, FMC63, human CD8α hinge and transmembrane domains, human 4-1BB (CD137) costimulatory domain, and CD3ζ signaling domain (Figure 1B). The plasmid vector contains a viral T2A self-cleaving peptide that permits ribosomal skipping and expression of a functionally inert tEGFR (Figure 1B). Tregs were transduced with retroviruses containing or lacking the hCAR19 construct; both constructs encoded the tEGFR reporter. Transduction efficiency was evaluated through tEGFR expression 4 days following retroviral transduction. hCAR19 and tEGFR Tregs each had approximately 30% tEGFR expression prior to enrichment (Figure 1D). Tregs were positively selected for tEGFR expression using MACS column purification to yield more than 85% EGFR^+^ Tregs with increased tEGFR mean fluorescence intensity (MFI) (Figure 1, C and D). MFI of CD25 and Foxp3 was comparable between nontransduced (NT), tEGFR control, and hCAR19-transduced Tregs (Figure 1E). The final Treg purity was at least 95% CD25^hi^Foxp3^+^ prior to experimental use (Figure 1F and Supplemental Figure 2).

### hCAR19 Tregs stimulated through their CAR have increased expression of canonical Treg antigens and demonstrate enhanced metabolic fitness.

Following stimulation with plate-bound recombinant hCD19 Fc protein for 48 hours, hCAR19 Tregs relative to tEGFR Tregs had enhanced expression of canonical Treg antigens associated with suppression, including cytotoxic T-lymphocyte antigen-4 (CTLA-4), T cell immunoreceptor with immunoglobulin and ITIM domain, neuropilin 1, and lymphocyte activation gene-3 (LAG-3) (Supplemental Figure 3, A–D). Also, the proliferation marker Ki67, the activation marker CD71, and the lineage-defining transcription factor Foxp3 were all expressed at higher levels on hCAR19 Tregs (Supplemental Figure 3, E–G). These data suggested that hCAR19 compared with tEGFR Tregs may respond more robustly in vivo in hCD19 hemizygous recipients (hCD19TG^Tg/0^), resulting in augmented immunosuppression and therapeutic protection.

In CAR19 human conventional T cells (Tcons), utilizing a 4-1BB intracellular costimulatory domain as compared with CD28 significantly enhances respiratory capacity, fatty acid oxidation, and mitochondrial biogenesis, all of which favor CD8^+^ T cell memory cells (37). In studies with CAR19 human Tregs, the same comparison shows a decrease in transcription of glycolysis genes (38). To determine whether CAR engagement could lead to differential metabolic states, hCAR19 and tEGFR murine Tregs were stimulated on an hCD19 Fc–coated plate for 48 hours prior to analysis. hCAR19 murine Tregs had significantly increased expression of carnitine palmitoyl transferase I (CPT1a), the rate limiting enzyme for fatty acid oxidation, and glucose transporter 1 (Glut1), as compared with tEGFR Tregs (Supplemental Figure 4, A and B). Seahorse mitochondrial and glycolytic stress tests were performed to further explore the involvement of oxidative phosphorylation and glycolysis by hCAR19 murine Tregs. hCAR19 Tregs had increased basal and maximal respiration, as well as spare respiratory capacity, compared with tEGFR Tregs (Supplemental Figure 4, C and D), along with a significant increase in glycolysis, glycolytic capacity, and glycolytic reserve (Supplemental Figure 4, E and F). Together, these data show hCAR19 murine Treg stimulation by its cognate antigen results in increased energetic capacity, as evidenced by higher oxidative phosphorylation and glycolysis to support immunosuppressive functions.

### hCAR19 Tregs deplete hCD19 B cells and prevent systemic toxicity.

**Figure 2:**
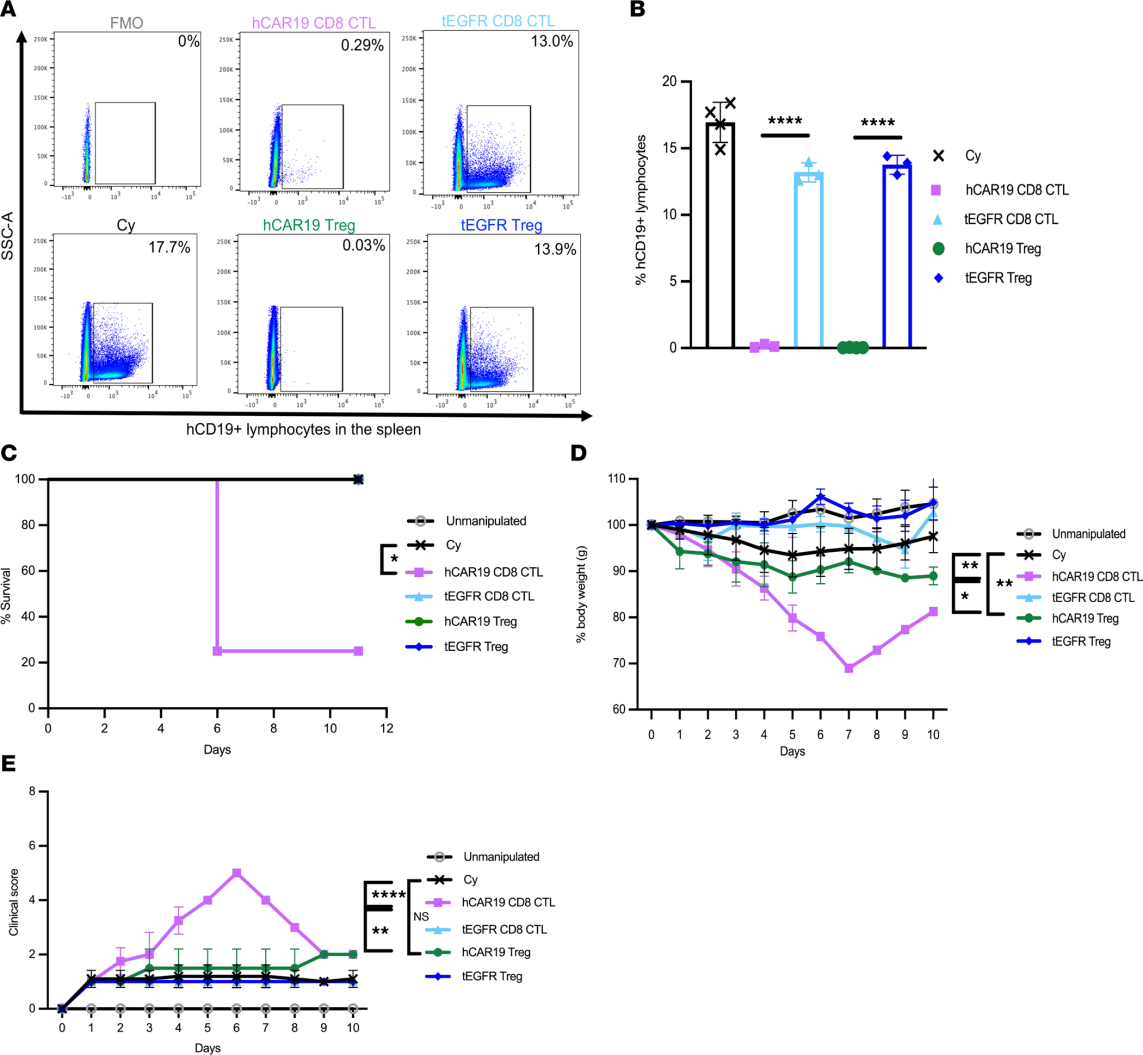
hCAR19 Tregs deplete hCD19 B cells and prevent systemic toxicities in a syngeneic mouse model. hCD19TG^Tg/0^ recipient mice injected with 300 mg/kg of cyclophosphamide (Cy) a day prior to adoptive cell transfer (ACT) with C57BL/6 hCAR19 or tEGFR Tregs, or hCAR19 or tEGFR CD8^+^ T cells (CTL). (**A**) Representative flow cytometry plots and quantification (**B**) of hCD19^+^ lymphocytes in the spleen on day 5 after ACT. Cy, *n* = 4; hCAR19 CD8^+^ CTLs, *n* = 3; tEGFR CD8^+^ CTLs, *n* = 3; hCAR19 Tregs, *n* = 4; tEGFR Tregs, *n* = 3. Data are representative from 2 independent experiments. (**C**) Survival. (**D**) Percentage body weight. (**E**) Clinical scores. Unmanipulated, *n* = 4; Cy, *n* = 4; hCAR19 CD8^+^ CTLs, *n* = 4; tEGFR CD8^+^ CTLs, *n* = 3; hCAR19 Tregs, *n* = 3; tEGFR Tregs, *n* = 3. Data are representative of 3 independent experiments. Statistics are shown on day 5 after ACT. Student’s *t* test with Bonferroni correction for multiple comparison was used for statistical analysis. Log rank test was used to analyze survival curves. Error bars indicate the standard deviation of the mean. *: *P* < 0.5; **: *P* < 0.01; ****: *P* < 0.0001.

To assess whether hCAR19 murine Tregs would induce B cell aplasia, we utilized our previously published syngeneic mouse model in which hCD19TG^Tg/0^ recipients are treated with cyclophosphamide (Cy) prior to CAR T cell infusion. In this model, once hCAR19 murine cytotoxic T lymphocytes (CTLs) are infused, recipients develop B cell aplasia associated with systemic toxicity and a high degree of lethality by day 10 postinfusion (36). hCD19TG^Tg/0^ recipients infused with hCAR19 murine Tregs or hCAR19 murine CD8^+^ CTLs had 0.03% and 0.29% hCD19 B cells on day 5 after adoptive cell transfer (ACT), respectively, whereas hCD19TG^Tg/0^ recipients treated with Cy only had 17.7% splenic B cells (Figure 2, A and B). In contrast, tEGFR murine Treg– and tEGFR murine CD8^+^ CTL–treated hCD19TG^Tg/0^ mice had 13.9% and 13.0% hCD19 splenic B cells. On day 10 after ACT, hCAR19 CD8^+^ CTL–treated hCD19TG^Tg/0^ mice had 80% lethality compared with 0% lethality in the hCAR19 Treg group (*P* < 0.05, survival; Figure 2C). Additionally, hCD19TG^Tg/0^ recipients of hCAR19 CD8^+^ CTLs had 30% mean body weight loss compared with less than 10% in mice that received hCAR19 murine Tregs or either tEGFR subset (*P* < 0.05, day 5, Figure 2D). Furthermore, hCAR19 murine CD8^+^ CTL–treated hCD19TG^Tg/0^ mice had mean clinical scores that peaked at 5 on day 6 after ACT, while hCAR19 Treg–, tEGFR CD8^+^ CTL–, or tEGFR Treg–treated hCD19TG^Tg/0^ mice had consistently lower clinical scores of at least 2, comparable to Cy only treated mice in the 8 days after ACT (Figure 2E). These results demonstrate that hCAR19 murine Tregs cause B cell aplasia without significant systemic toxicity or lethality in hCD19TG^Tg/0^ recipients, in contrast to the severe side effects and lethality of hCAR19 murine CD8^+^ CTLs.

We also evaluated whether hCAR19 or tEGFR Tregs could suppress the systemic toxicity induced by hCAR19 CD8^+^ CTLs. Using a 2:1 ratio of hCAR19 CD8^+^ CTLs to hCAR19 or tEGFR Tregs, we found that Treg treatment fully abrogated lethality in this model and significantly reduced clinical scores and weight loss (Supplemental Figure 5). hCAR19 Treg–treated mice compared with tEGFR Treg–treated mice demonstrated significantly higher mean weights that were maintained throughout the observation period, though there was no difference in clinical scores between these 2 cohorts, both of which were lower than hCAR19 CD8^+^ CTLs (Supplemental Figure 5, B and C). These data suggest hCAR19 Tregs can be coinfused with hCAR19 CD8^+^ CTLs to reduce systemic toxicities.

### hCAR19 Tregs control hCD19 TBL12-luciferase growth in vivo.

**Figure 3:**
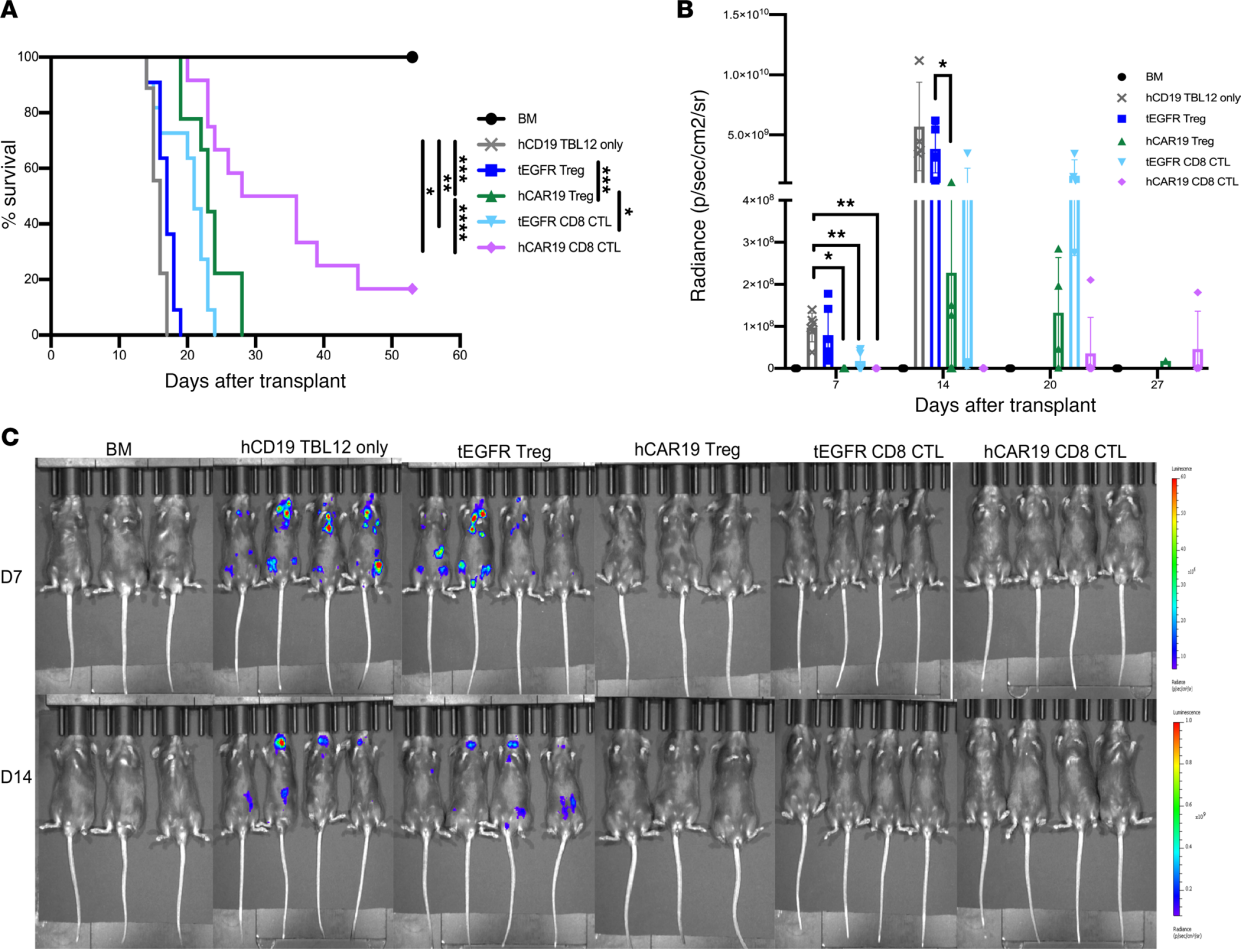
hCAR19 Tregs have antitumor responses in the absence of Tcons in a syngeneic tumor model. Survival (**A**) and average radiance (**B**) of hCD19TG^Tg/0^ mice after undergoing a lethal irradiation prior to receiving C57BL/6 bone marrow (BM), BM with hCD19 TBL12^luc^ cells, or BM with hCD19 TBL12^luc^ cells and either tEGFR or hCAR19 Tregs or CTLs. CTLs in this experiment were CD8^+^ T cells. Survival: BM, *n* = 10; hCD19 TBL12^luc^, *n* = 9; tEGFR Tregs, *n* = 11; hCAR19 Tregs, *n* = 9; tEGFR CTLs, *n* = 11; hCAR19 CTLs, *n* = 12. Data are pooled from 2 independent experiments. Average radiance: BM, *n* = 6; hCD19 TBL12^luc^, *n* = 6; tEGFR Tregs, *n* = 7; hCAR19 Tregs, *n* = 6; tEGFR CTLs, *n* = 6; hCAR19 CTLs, *n* = 7. Only 3–4 mice were imaged per group. Data are pooled from 2 independent experiments. (**C**) Representative images of hCD19TG^Tg/0^ mice on day 7 and 14 after syngeneic HSCT. D7 radiance scale: 10-60 x 10^6^; D14 radiance scale: 0.2-1 x 10^9^. Student’s *t* test with Bonferroni correction for multiple comparison was used for statistical analysis. Log rank test was used to analyze survival curves. Error bars indicate the standard deviation of the mean. *:<0.5; **: <0.01; ***:<0.001; ****:<0.0001.

To evaluate whether hCAR19 murine Tregs had the capacity to eliminate hCD19 TBL12-luciferase (TBL12^luc^) tumor cells in vivo, hCD19TG^Tg/0^ mice were lethally irradiated, then transplanted with 5 × 10^6^ C57BL/6 T cell–depleted (TCD) bone marrow (BM). Cohorts were injected with 10^4^ hCD19 TBL12^luc^ alone or no cells and hCAR19 Tregs, hCAR19 CD8^+^ CTLs, tEGFR Tregs, or tEGFR CD8^+^ CTLs. hCD19TG^Tg/0^ recipients treated with either tEGFR Tregs or tEGFR CD8^+^ CTLs had 100% lethality by day 18 and day 24, respectively, significantly faster than those receiving hCAR19 Tregs that required 28 days for uniform lethality. The best survival rate was observed in hCD19TG^Tg/0^ mice treated with hCAR19 CD8^+^ CTLs that had an 83.3% lethality rate by week 7 (Figure 3A), significantly better than all other cohorts receiving immune cell therapy.

On day 7 after transplantation, hCD19TG^Tg/0^ mice injected with hCD19 TBL12^luc^ cells and tEGFR Tregs or tEGFR CD8^+^ CTLs had detectable tumor by bioluminescence imaging (BLI). By day 14, only mice receiving hCD19 CD8^+^ CTLs had no detectable tumor (Figure 3, B and C). Notably, hCAR19 Treg– and tEGFR CD8^+^ CTL–treated mice had significant tumor growth on day 14 and 20, ultimately leading to death in these mice before day 30 (Figure 3, A and B). tEGFR Treg–treated mice had a comparable mortality curve and tumor growth pattern to mice injected with hCD19 TBL12^luc^ only, where mice began to have measurable tumor growth as early as day 7 after transplant and all succumbed to disease prior to day 20 (Figure 3, A–C). hCAR19 CD8^+^ CTLs largely controlled tumor growth during the first 3 weeks but ultimately had tumor outgrowth that led to 17% survival (Figure 3, A–C). Overall, these data demonstrate hCAR19 Tregs can control hCD19 TBL12^luc^ growth in hCD19TG^Tg/0^ mice early after a syngeneic transplant.

### hCAR19 Tregs kill hCD19 TBL12^luc^ tumor cells in a perforin-dependent, GzB-independent manner.

**Figure 4:**
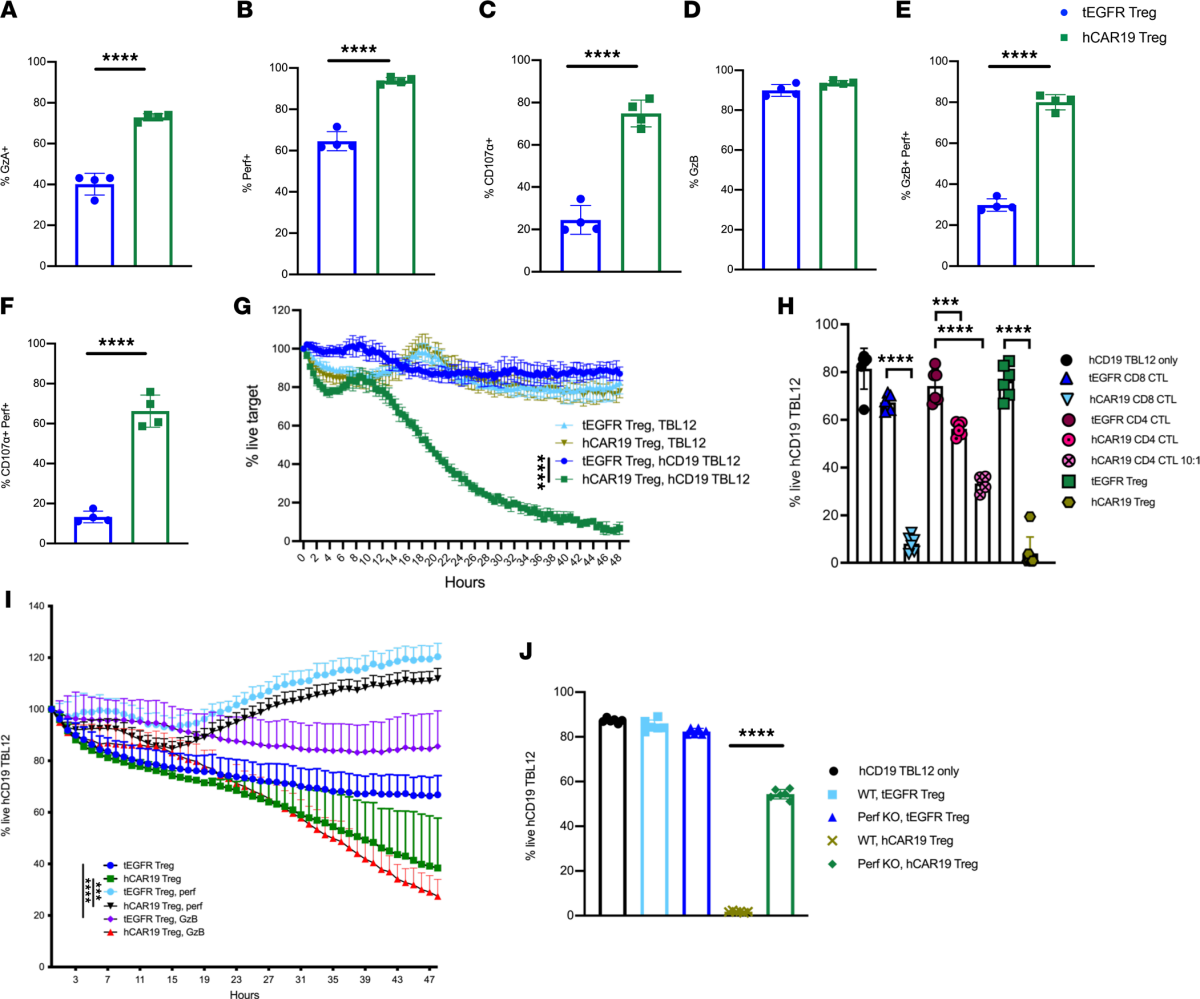
hCAR19 Tregs have increased expression of killing markers after antigen-specific activation and engage in in vitro killing of hCD19 TBL12. (**A**–**F**) Frequency of hCAR19 or tEGFR Tregs expressing granzyme A (GzA), perforin (perf), CD107α, and granzyme B (GzB) after 48-hour stimulation in an hCD19 Fc–coated plate. hCAR19 Tregs, *n* = 4; tEGFR Tregs, *n* = 4. (**G**) IncuCyte in vitro killing assay with TBL12 and hCD19 TBL12^luc^ tumor cells stained with Far Red dye and the IncuCyte Caspase-3/7 green apoptosis dye. Effector-to-target (E/T) ratio used was 2:1. *n* = 3 for all groups. (**H**) A 48-hour flow cytometry in vitro killing assay using hCD19 TBL12^luc^ with sorted Foxp3^+^GFP^+^ Tregs the day of the experiment using a 5:1 E/T ratio in all groups except where noted to be 10:1. *n* = 6 for all groups. (**I**) IncuCyte in vitro killing assay using the perforin inhibitor concanamycin A (CMA) and GzB inhibitor Z-AAD-CMK with hCD19 TBL12^luc^ tumor. E/T ratio used was 2:1. *n* = 5 for all groups, except hCAR19 Treg and hCAR19 Treg GzB had *n* = 3. (**J**) A 48-hour flow cytometry in vitro killing assay using hCD19 TBL12^luc^ at an E/T ratio of 5:1. *n* = 6 for all groups. Statistics for IncuCyte experiments were done at the 48-hour time point. Data in this figure are representative from 2 independent experiments. Student’s *t* test with Bonferroni correction for multiple comparisons was used for statistical analysis. Unpaired *t* test (1 tailed) was used for single comparisons. Error bars indicate the standard deviation of the mean. ***:<0.001; ****:<0.0001.

To better understand the mechanistic underpinnings of hCAR19 Treg–mediated elimination of both hCD19^+^ B cells and hCD19 TBL12^luc^ growth in vivo, we pursued phenotypic and functional analyses related to their cytolytic potential. Following stimulation of hCAR19 Tregs in an hCD19 Fc–coated plate, we found that hCAR19 Tregs compared with tEGFR Tregs had a significant increase in the frequency and MFI of granzyme A (GzA), GzB, perforin, and CD107α (Figure 4, A–F; and Supplemental Figure 6, A–D). We next tested the killing potential of hCAR19 and tEGFR Tregs by serial measurements over 48 hours using the IncuCyte Immune Cell Killing Assay. Tregs were cocultured in the presence of either TBL12 or hCD19 TBL12^luc^ cells labeled with Far Red Dye and the IncuCyte Caspase-3/7 green apoptosis dye. We found hCAR19 Tregs engaged in antigen-specific killing, with only 7% live hCD19 TBL12^luc^ cells remaining after 48 hours of coculture compared with 78% live TBL12 cells (Figure 4G). In contrast, wells containing tEGFR Tregs had 87% live hCD19 TBL12^luc^ and 82% live TBL12 (Figure 4G).

We then assessed the expression of cytolytic molecules following anti-CD3/CD28 Dynabead stimulation used for retroviral transduction. hCAR19 and tEGFR CD8^+^ CTLs expressed comparable levels of GzA and GzB, whereas hCAR19 and tEGFR Tregs had negligible expression (Supplemental Figure 6E). While Fas was increased in Tregs compared with CTLs, perforin, FasL, and CD107α were comparably expressed (Supplemental Figure 6E). Next, to evaluate the killing potential of hCAR19 Tregs compared with hCAR19 CTLs, a flow cytometry killing assay was performed using Far Red–labeled hCD19 TBL12^luc^ cells at a 5:1 E/T ratio. hCAR19 and tEGFR Tregs were sorted after retroviral transduction to achieve the highest Treg purity and ensure killing was not associated with contaminating hCAR19 CD4^+^ CTLs. After a 48-hour coculture, tEGFR CD8^+^ CTLs had 67% live hCD19 TBL12^luc^ cells, while tEGFR CD4^+^ CTLs and tEGFR Tregs had 74% and 76%, respectively (Figure 4H and Supplemental Figure 6F). hCAR19 CD4^+^ CTLs engaged in killing of hCD19 TBL12^luc^ with 56% live cells at 5:1 E/T and 33.3% at the 10:1 E/T ratio (Figure 4H). In contrast, hCAR19 Tregs and hCAR19 CD8^+^ CTLs had 4% and 8% live hCD19 TBL12^luc^, respectively (Figure 4H and Supplemental Figure 6F). Over multiple in vitro flow killing assays using a 5:1 E/T ratio, hCAR19 Tregs averaged 1%–19% live hCD19 TBL12^luc^ cells, which was not significantly different from hCAR19 CD8^+^ CTLs, which had 1%–13% live hCD19 TBL12^luc^ cells (data not shown).

To assess the role of cytolytic pathways in hCAR19 Treg–mediated killing, concanamycin A (CMA) and Z-AAD-CMK inhibitors were used to inhibit the expression of perforin and GzB, respectively. hCAR19 Tregs killed hCD19 TBL12^luc^ in a perforin-dependent, GzB-independent manner (Figure 4I and Supplemental Figure 7). After 48-hour coculture, hCAR19 Tregs resulted in 38% live hCD19 TBL12^luc^, which was essentially unchanged to 27% live hCD19 TBL12^luc^ when Z-AAD-CMK, a GzB inhibitor, was added. In marked contrast, hCAR19 Treg coculture with a perforin inhibitor resulted in 112% live hCD19 TBL12^luc^ (Figure 4I). Findings were similar with tEGFR Tregs that showed no significant difference when cocultured with a GzB inhibitor, and background, nonspecific killing was abrogated when cocultured with a perforin inhibitor (Figure 4I and Supplemental Figure 7). Moreover, hCAR19 Tregs derived from perforin-knockout mice had an approximately 2.5-fold reduction in hCD19 TBL12^luc^ killing (Figure 4J). To investigate whether hCAR19 Tregs could engage in nonspecific killing of nearby Tcons in the presence of hCD19 B cells, hCAR19 and tEGFR Tregs were cocultured with Tcons in the presence of WT or hCD19TG^Tg/0^ antigen-presenting cells (APCs). After 72 hours, hCAR19 and tEGFR Tregs had comparable viability of CD4^+^ and CD8^+^ Tcons in the presence of WT or hCD19TG^Tg/0^ APCs (Supplemental Figure 8). These data show hCD19 CAR Tregs kill in antigen-dependent, perforin-dependent, GzB-independent manner.

### hCAR19 Tregs ameliorate aGVHD onset and severity without toxicity.

To ensure antigen would be present to activate hCAR19 Tregs early after allo-HSCT, we quantified hCD19 B cells in hCD19TG^Tg/0^ recipient mice after lethal irradiation and transplantation with BALB/c BM. Splenic and mesenteric lymph node (mLN) hCD19 B cell frequencies decreased by 19% and 9% on day 1 after transplantation, whereas by day 7 splenic and mLN B cell frequencies were decreased by 86% and 58%, respectively (Supplemental Figure 9). There was a further reduction by day 14 such that both spleen and mLN had ≥86% fewer cells compared with nontransplanted hCD19TG^Tg/0^ mice (Supplemental Figure 9). Additionally, peripheral blood hCD19 B cells decreased rapidly by approximately 75% on day 1 and had negligible detection by day 14 (Supplemental Figure 9). These data demonstrated hCD19 B cells are present in the spleen and mLNs of hCD19TG^Tg/0^ mice through day 7 and up to day 14 in the mLNs, providing antigen necessary for hCAR19 Tregs to be activated and engage in immunosuppressive functions.

**Figure 5:**
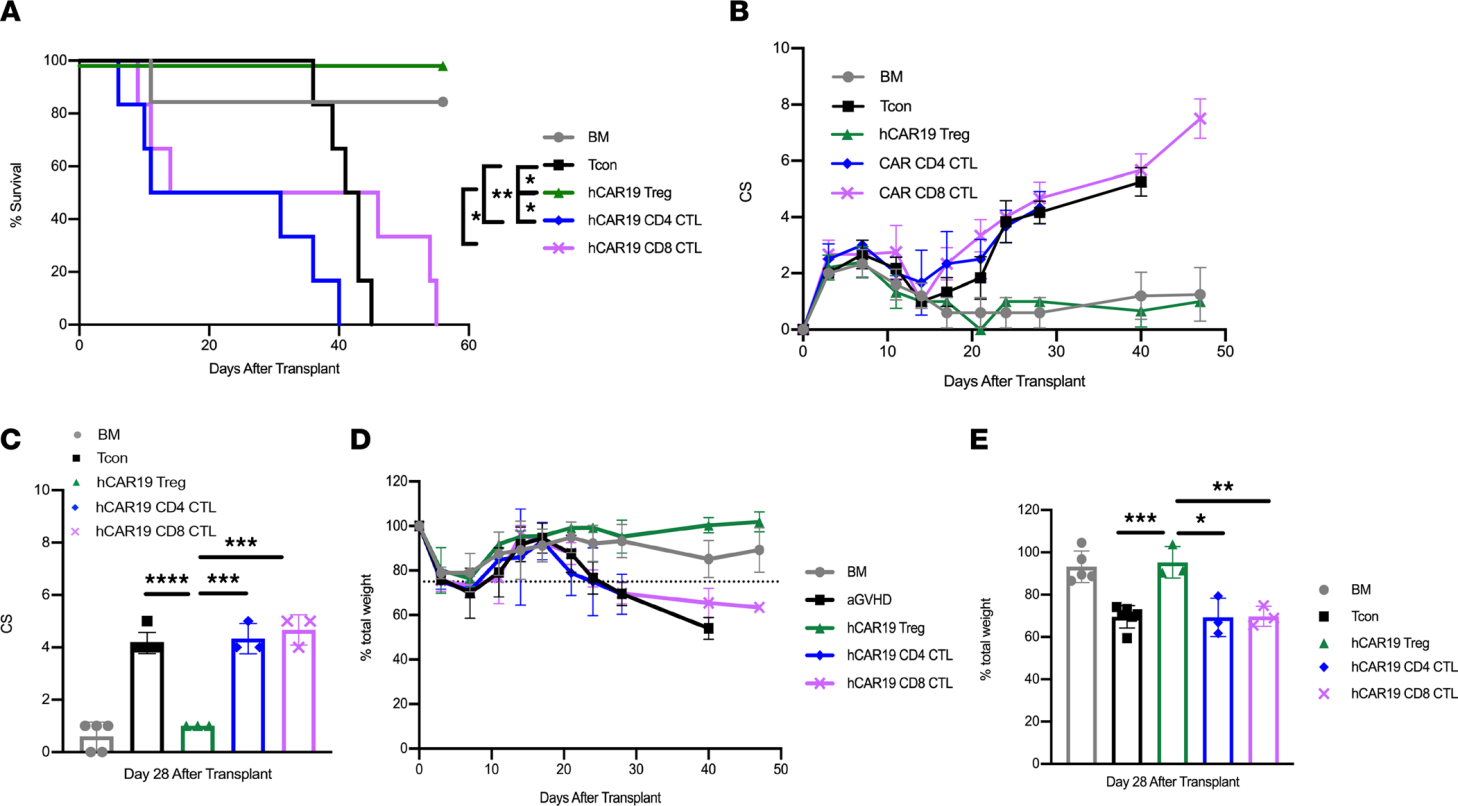
hCAR19 Tregs reduce aGVHD severity and mortality relative to hCAR19 CTLs. (**A**) Survival for hCD19TG^Tg/0^ recipient mice after undergoing a lethal irradiation prior to receiving BALB/c BM only; BM with Tcons; or BM with Tcons and hCAR19 Tregs, hCAR19 CD4^+^ CTLs, or hCAR19 CD8^+^ CTLs. BM, *n* = 6; Tcons, *n* = 6; hCAR19 Tregs, *n* = 6; hCAR19 CD4^+^ CTLs, *n* = 6; hCAR19 CD8^+^ CTLs, *n* = 6. Data are representative from 2 independent experiments. (**B**) Clinical GVHD scores: 0, no disease; 10, severe disease. (**C**) Clinical scores quantified on day 28. (**D**) Percentage body weight. Dotted line represents 75% body weight. (**E**) Percentage body weight quantified on day 28. Student’s *t* test with Bonferroni correction for multiple comparisons was used for statistical analysis. Log rank test was used to analyze survival curves. Error bars indicate the standard deviation of the mean. *:<0.5; **: <0.01; ***:<0.001; ****:<0.0001.

We next asked if hCAR19 Tregs conferred higher aGVHD protection as compared with hCD19 CAR T cells, as the addition of T cells could accelerate aGVHD lethality due to its T cell–mediated pathogenesis (3). To investigate this question, hCD19TG^Tg/0^ mice were lethally irradiated a day prior to receiving BALB/c BM only; BM with Tcons to induce aGVHD; or BM with Tcons and hCAR19 Tregs, hCAR19 CD4^+^ CTLs, or hCAR19 CD8^+^ CTLs on day 0 of allo-HSCT. hCAR19 Tregs significantly suppressed aGVHD as shown by the absence of lethality and with clinical scores and weights overlapping with that of the BM only group (Figure 5, A–E). In contrast, hCAR19 CD4^+^ CTLs significantly exacerbated disease, causing faster mortality compared with the aGVHD only group (Figure 5A). hCAR19 CD4^+^ and CD8^+^ CTL–treated mice showed increased weight loss and clinical scores similar to those of the aGVHD group (Figure 5, B–E). While aGVHD mice fully succumbed to disease by day 45 after transplant, aGVHD mice treated with hCAR19 CD8^+^ CTLs or hCAR19 CD4^+^ CTLs died by day 55 and day 40, respectively (Figure 5A).

**Figure 6:**
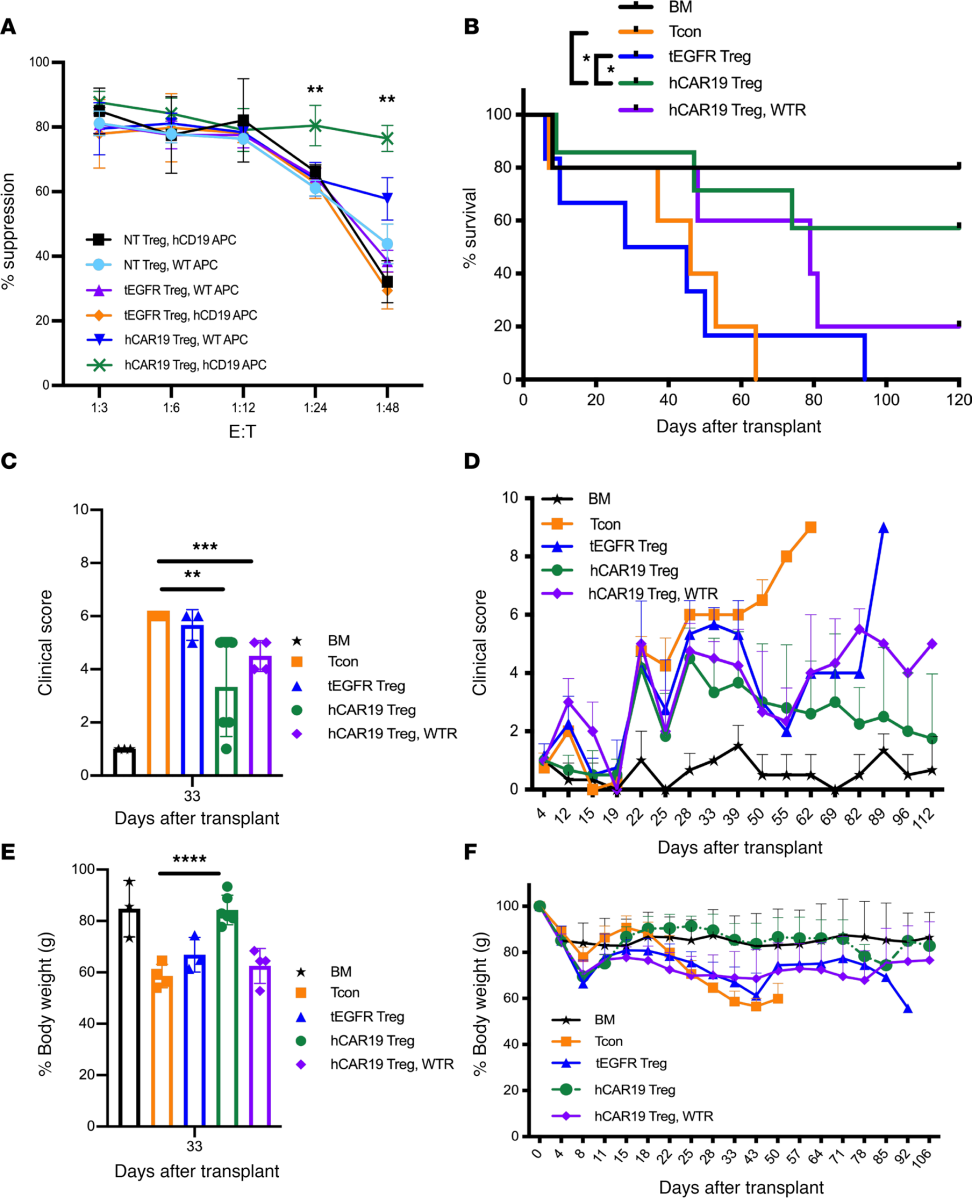
hCAR19 Tregs reduce aGVHD severity and improve overall survival in an hCD19-dependent manner. (**A**) Percentage suppression by NT, tEGFR, or hCAR19 Tregs of CD8^+^ T cells in the presence of WT or hCD19-containing B cells. (**B**) Survival for hCD19TG^Tg/0^ recipient mice after undergoing a lethal irradiation prior to receiving 5 × 10^6^ BALB/c BM; BM with 2.5 × 10^6^ Tcons; or BM with Tcons and either 1.25 × 10^6^ hCAR19 or tEGFR Tregs; or WT C57BL/6 recipient mice receiving BALB/c BM with Tcons and hCAR19 Tregs (hCAR19 Treg, WTR). BM, *n* = 5; Tcons, *n* = 8; tEGFR Tregs, *n* = 6; hCAR19 Tregs, *n* = 6; hCAR19 Treg, WTR, *n* = 5. Data are representative from 4 independent experiments. (**C** and **D**) Clinical GVHD scores: 0, no disease; 10, severe disease. (**C**) Clinical GVHD scores quantified on day 33. (**E**) Percentage body weight quantified on day 33. (**F**) Percentage body weight. Student’s *t* test with Bonferroni correction for multiple comparisons was used for statistical analysis of weights and clinical scores. Log rank test was used to analyze survival curves. Error bars indicate the standard deviation of the mean. *:<0.5; **: <0.01; ***:<0.001; ****:<0.0001.

Next, we evaluated the contribution of hCD19 B cells to hCAR19 Treg suppressive function. We cocultured NT, tEGFR, and hCAR19 Tregs at multiple E/T ratios with CD25-depleted Tcons labeled with CellTrace Violet (CTV) dye to track their proliferation. Tregs and Tcons were isolated from BALB/c mice, whereas APCs were isolated from C57BL/6 background mice in order to mimic the allo-setting in our in vivo GVHD model. We found that hCAR19 Tregs had significantly higher suppressive function at the 1:24 and 1:48 E/T ratios when cocultured with hCD19 B cell–containing APCs as compared with WT APCs (Figure 6A). There were no significant differences in suppression among NT and tEGFR Tregs cocultured with WT or hCD19 B cell–containing APCs and hCAR19 Tregs with WT APCs (Figure 6A).

We then tested whether hCAR19 Tregs were dependent on hCD19 B cell expression to enhance aGVHD suppression. hCD19TG^Tg/0^ mice were lethally irradiated 1 day prior to receiving BALB/c BM only, BM with Tcons to induce aGVHD, or BM with Tcons and either hCAR19 or tEGFR Tregs (1.25 × 10^6^) on day 0 of allo-HSCT. An additional cohort consisted of WT C57BL/6 recipient mice to evaluate hCAR19 Treg efficacy in the absence of hCD19 antigen. To assess the differences between hCAR19 and tEGFR Tregs, we infused suboptimal GVHD prophylaxis Treg doses at a 1:2 E/T ratio. hCAR19 Tregs significantly improved aGVHD survival compared with tEGFR Tregs (Figure 6B), with an overall survival of 57% versus 0% on day 120 after transplant. hCAR19 Tregs infused into WT mice resulted in only 20% survival, suggesting a role of hCD19 B cells in supporting hCAR19 Treg suppression of aGVHD. These data suggest hCD19 antigen is necessary for hCAR19 Treg–enhanced function when compared with tEGFR Tregs (Figure 6B). Consistent with survival data, hCD19TG^Tg/0^ recipients treated with hCAR19 Tregs had significantly reduced clinical scores (Figure 6, C and D) and maintained body weights comparable to those of BM-only recipients (Figure 6, E and F). hCD19TG^Tg/0^ mice given tEGFR Tregs and WT mice given hCAR19 Tregs did not improve mean body weights over those of the aGVHD group (Figure 6, E and F). Overall, hCAR19 Tregs compared with tEGFR Tregs significantly reduced the onset and severity of aGVHD in an hCD19 B cell–dependent manner.

### hCAR19 Tregs have greater expansion early after allo-HSCT and demonstrate enhanced suppression of colonic Tcons compared with tEGFR Tregs.

**Figure 7:**
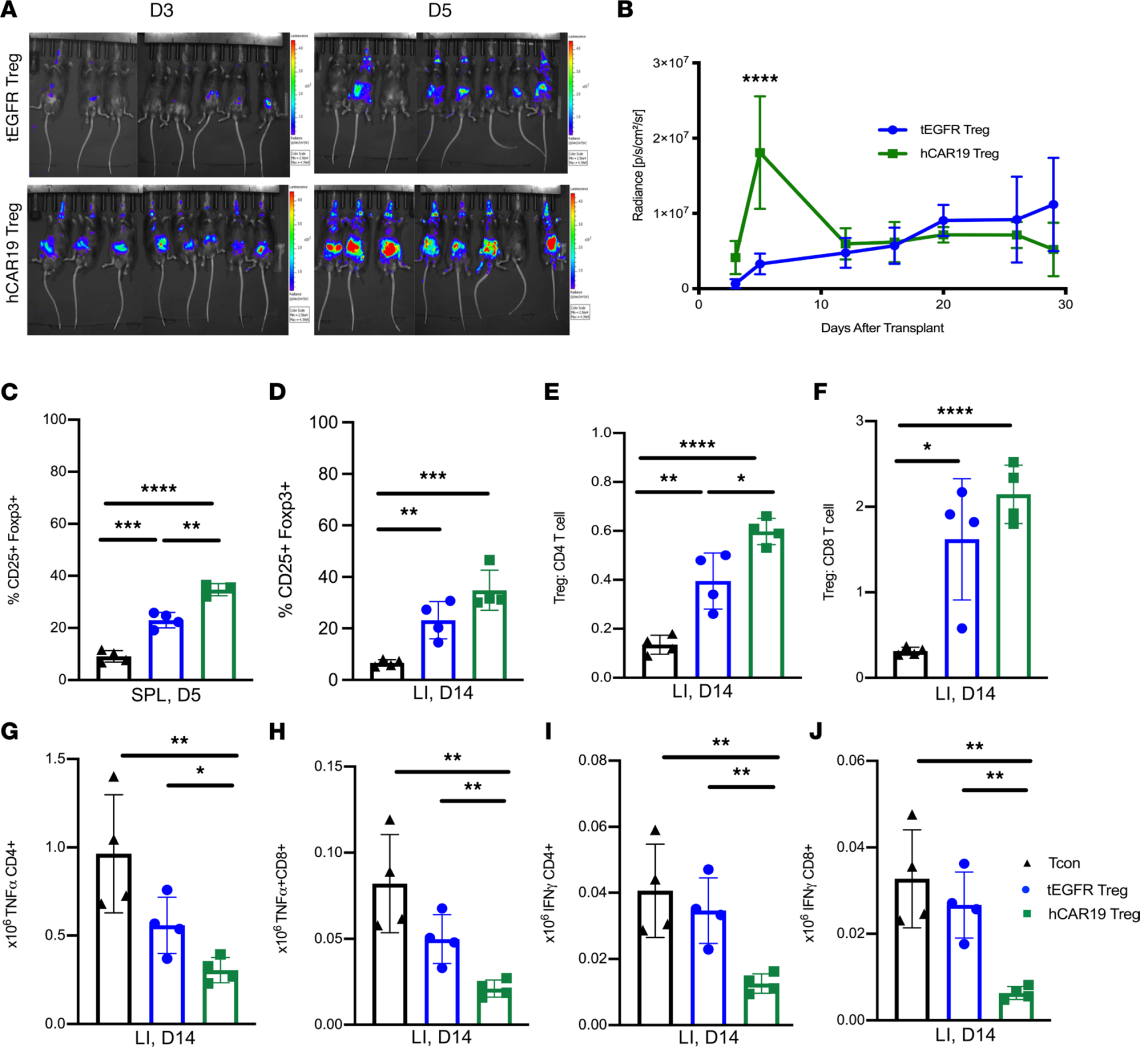
hCAR19 Tregs have increased expansion and suppression of activated T cells in the colon following allo-HSCT. (**A**) Images of hCD19TG^Tg/0^ mice on day 3 and 5 after receiving BALB/c BM with Tcons and luciferase^+^ hCAR19 or tEGFR Tregs. Radiance scale of 1.0-4.0 x 10^5^. (**B**) Average radiance of luciferase^+^ hCAR19 or tEGFR Tregs from day 3 to 30 after allo-HSCT. tEGFR Tregs, *n* = 8; hCAR19 Tregs, *n* = 8. (**C**) Frequency of CD25^+^Foxp3^+^ Tregs in the spleen on day 5 after allo-HSCT. Tcons, *n* = 4; tEGFR Tregs, *n* = 4; hCAR19 Tregs, *n* = 3. (**D**–**J**) Colon was harvested on day 14 after allo-HSCT in hCD19TG^Tg/0^ recipient mice that received BALB/c BM with Tcons, or BM with Tcons and tEGFR or hCAR19 Tregs. (**E** and **F**) Treg to CD4^+^ and CD8^+^ T cell ratios. (**G**–**J**) Absolute number of TNF-α^+^ and IFN-γ^+^ CD4^+^ and CD8^+^ T cells. Tcons, *n* = 4; tEGFR Tregs, *n* = 4; hCAR19 Tregs, *n* = 4. SPL, spleen; LI, large intestine. Data from all experiments are representative from 2 independent experiments. Student’s *t* test with Bonferroni correction for multiple comparison was used for statistical analysis. Error bars indicate the standard deviation of the mean. *:<0.5; **: <0.01; ***:<0.001; ****:<0.0001.

To evaluate how hCAR19 Tregs reduce aGVHD lethality, we first quantified Treg expansion in vivo using BLI of luciferase-expressing Tregs. hCD19TG^Tg/0^ mice treated with hCAR19 Tregs compared with tEGFR Tregs showed significantly improved BLI signal by day 5 after allo-HSCT (Figure 7, A and B). Radiance in the tEGFR Treg group slowly increased over time, equalized to that of hCAR19 Tregs by day 14, and remained comparable until the end of the observation period, which was day 28 (Figure 7B). Results of BLI were confirmed by enumerating splenic Tregs. A higher frequency of CD25^+^Foxp3^+^ Tregs was observed in hCD19TG^Tg/0^ mice treated with hCAR19 Tregs compared with tEGFR Tregs on day 5 after allo-HSCT (Figure 7C). Additionally, hCAR19 Tregs compared with tEGFR Tregs caused a greater reduction in the frequency of day 5 splenic CD4^+^ and CD8^+^ Tcons, while both Treg groups comparably decreased the frequency of CD11c-expressing monocytes and DCs (Supplemental Figure 10).

Next, we evaluated hCAR19 Treg homing and protection of the gastrointestinal tract (GIT), as the GIT is the target organ associated with the highest aGVHD morbidity and mortality (3). Both hCAR19 and tEGFR Tregs homed to the colon by day 14 after allo-HSCT (Figure 7D). hCAR19 Tregs significantly increased the Treg/CD4^+^ T cell and Treg/CD8^+^ T cell ratios (Figure 7, E and F). These results were consistent with the finding that hCAR19 Tregs more potently reduced the number of proinflammatory TNF-α– and IFN-γ–producing, colonic, CD4^+^ and CD8^+^ T cells (Figure 7, G–J). These data suggest that hCAR19 compared with tEGFR Tregs have enhanced Treg expansion early after allo-HSCT, leading to increased Treg/Tcon ratios and decreased numbers of Tcons in the colon, a key aGVHD target organ.

### hCAR19 Tregs maintain GVT responses in the allo-HSCT setting.

**Figure 8:**
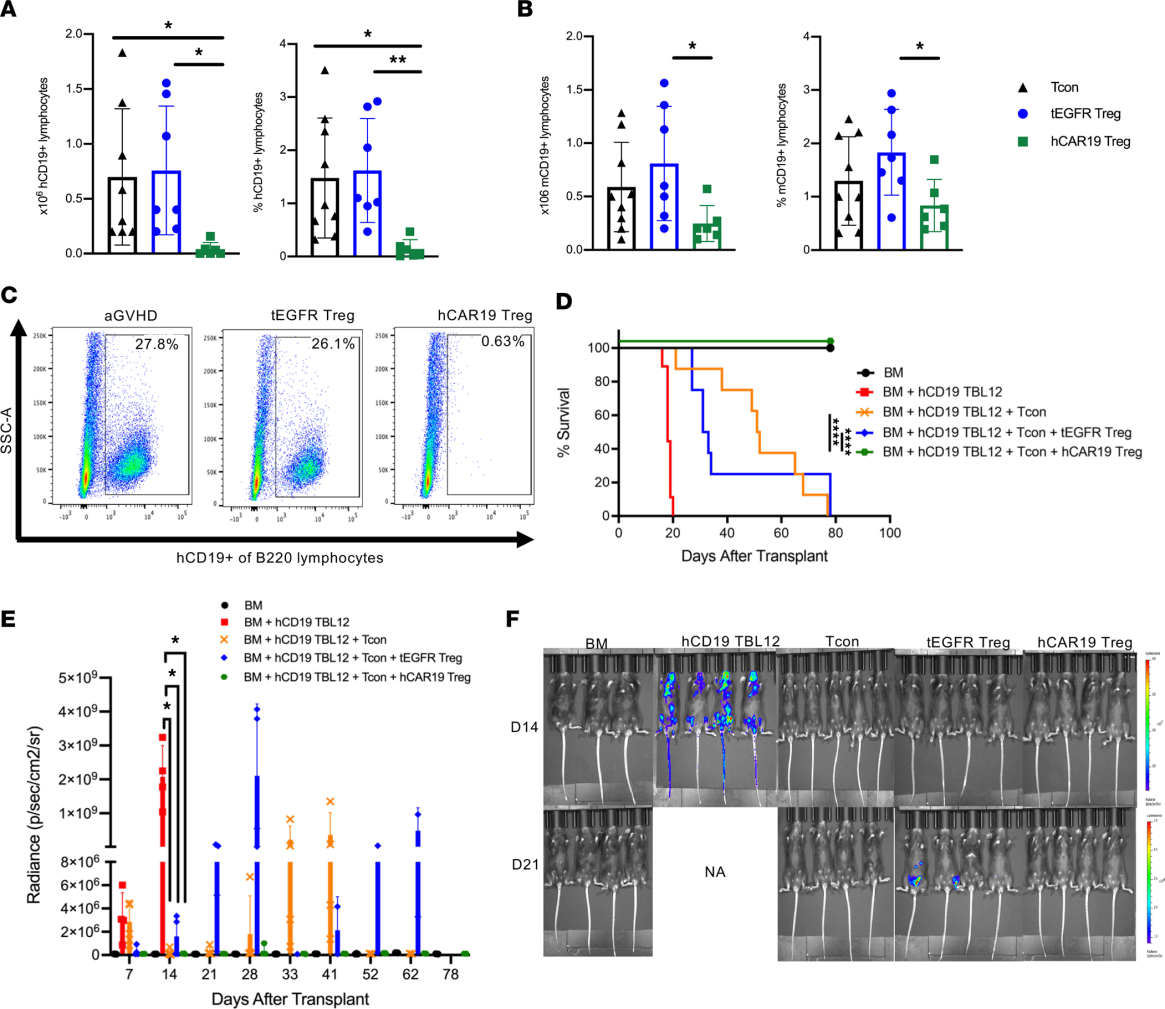
hCAR19 Tregs maintain GVT responses. (**A**) Absolute number and frequency of hCD19^+^ and mouse CD19^+^ (mCD19^+^) (**B**) lymphocytes in the spleen on day 5 of hCD19TG^Tg/0^ recipient mice after undergoing a lethal irradiation prior to receiving BALB/c BM with Tcons or BM with Tcons and either hCAR19 or tEGFR Tregs. Data are pooled from 2 independent experiments. Tcons, *n* = 8; tEGFR Tregs, *n* = 7; hCAR19 Tregs, *n* = 6. (**C**) Representative flow plots demonstrating hCD19^+^ lymphocyte depletion in hCAR19 Treg–treated mice on day 5 after allo-HSCT. hCD19 lymphocytes were gated from live B220 cells. (**D**) Survival of hCD19TG^Tg/0^ recipient mice after undergoing a lethal irradiation prior to receiving BALB/c BM, BM with hCD19 TBL12, BM with hCD19 TBL12 with Tcons, or BM with hCD19 TBL12 and Tcons and either hCAR19 or tEGFR Tregs. Data are representative from 2 independent experiments. BM, *n* = 7; hCD19 TBL12^luc^, *n* = 9; Tcons, *n* = 8; tEGFR Tregs, *n* = 8; hCAR19 Tregs, *n* = 9. (**E**) Average radiance of hCD19 TBL12^luc^. Average radiance: BM, *n* = 4; hCD19 TBL12^luc^, *n* = 4; tEGFR Tregs, *n* = 4; hCAR19 Tregs, *n* = 4. Only 3–5 mice were imaged per group. Data are representative from 2 independent experiments. (**F**) Images from day 14 and 21 after allo-HSCT. Student’s *t* test with Bonferroni correction for multiple comparisons was used for statistical analysis. Log rank test was used to analyze survival curves. Error bars indicate the standard deviation of the mean. *:<0.5; **: <0.01; ****:<0.0001.

Increased aGVHD suppression and reduction of proinflammatory cytokine–producing Tcons by hCAR19 Tregs could interfere with the GVT response. Therefore, we evaluated whether hCAR19 Tregs had the capacity to deplete hCD19 B cells in hCD19TG^Tg/0^ recipient mice in the allo-HSCT setting. Mice transplanted with BM, Tcons, and hCAR19 Tregs had significantly lower frequencies and absolute numbers of hCD19 B cells as compared with those receiving tEGFR Tregs or no Tregs (Figure 8, A–C). To determine whether hCAR19 Tregs would interfere with the GVT response, hCD19TG^Tg/0^ recipients were coinfused with hCD19 TBL12^luc^ cells on day 0. hCD19TG^Tg/0^ mice that received BM and hCD19 TBL12^luc^ cells had 100% mortality related to lymphoma by day 20 (Figure 8D). In contrast, mice receiving BM with hCD19 TBL12^luc^ and Tcons had 37.5% death by day 50 related to lymphoma with late deaths that occurred by day 80 related to aGVHD (Figure 8D). Mice treated with tEGFR Tregs had 75% death related to lymphoma by day 40, with the remainder fully succumbing to aGVHD by day 80 (Figure 8D). In striking contrast, mice treated with BM, Tcons, hCD19 TBL12^luc^ cells, and hCAR19 Tregs, had 100% survival (Figure 8D). In support of the survival data, mice injected with hCD19 TBL12^luc^ in the absence of Tcons had measurable tumor growth on day 7, and significant expansion by day 14, providing evidence that the early deaths in this group were lymphoma related (Figure 8E). All mice except 1 treated with hCD19 TBL12^luc^ and Tcons demonstrated tumor growth control up to day 28, whereas the addition of tEGFR Tregs led to measurable tumor growth starting on day 7 after allo-HSCT (Figure 8, E and F). hCAR19 Tregs potently suppressed tumor growth throughout the entirety of this study (Figure 8, E and F). These data demonstrate hCAR19 Tregs, unlike tEGFR Tregs, not only potently suppressed aGVHD but also maintained GVT by potentiating antitumor responses.

## Discussion

Here, we used a syngeneic mouse model to demonstrate that hCAR19 Tregs depleted hCD19TG^Tg/0^ recipient hCD19 B cells without inducing systemic toxicities, as is seen with hCAR19 CD8^+^ CTLs. In this same model, hCAR19 Tregs controlled hCD19 TBL12^luc^ tumor growth in vivo, leading to a marked improvement in survival as compared with mice injected with tumor cells alone or tumor cells with tEGFR Tregs. In vitro killing assays demonstrated hCAR19 Tregs used the perforin pathway to mediate antigen-specific killing. In a fully MHC-mismatched allo-HSCT murine model, hCAR19 Tregs suppressed aGVHD whereas hCAR19 CD8^+^ CTLs failed to provide therapeutic protection. The presence of hCD19 B cells in recipient mice was necessary for the enhanced suppressive function of hCAR19 Tregs compared with control tEGFR Tregs in our aGVHD model. We showed that hCD19TG^Tg/0^ mice treated with hCAR19 Tregs had hCD19 B cell depletion as early as day 5 after allo-HSCT; nonetheless, there was significant Treg expansion on day 5 after allo-HSCT in hCAR19 Treg–treated mice compared with tEGFR Treg–treated hCD19^TG/0^ mice, with significantly improved suppression of proinflammatory cytokine–producing T cells in the gut (Figure 7). Our findings are consistent with what is known in the literature, which is that to have effective GVHD suppression, it must occur early after allo-HSCT to target the key GVHD induction stages, such as alloantigen priming (3). Importantly, the hCD19 B cell killing capacity of hCAR19 Tregs provided an advantage to obtaining a GVT response directed against hCD19 TBL12^luc^. These studies report for the first time to our knowledge an hCAR Treg therapy that suppresses aGVHD and kills antigen-expressing tumor cells to prevent hCD19 lymphoma recurrence without causing systemic toxicities.

The efficacy of CAR T cell therapy in treating B cell malignances has been hindered by severe toxicities resulting from high levels of inflammatory cytokines (e.g., CRS) (32). While most patients with CRS experience mild symptoms, some cases are severe or life-threatening. A primary treatment for CRS is the IL-6Rα antagonist tocilizumab (33). Corticosteroids are used for resistant CRS cases and for CAR-related neurological complications (32). Some reports indicate that corticosteroids can negatively affect CAR T cell persistence and function (39, 40), whereas other reports do not link steroids to poorer outcomes (41). Nonetheless, CAR T cell therapies that do not induce deleterious, systemic inflammation may prove more efficacious than existing ones.

Unlike CAR CD8^+^ CTLs, CAR Tregs produced virtually no inflammatory cytokines following antigen activation, suggesting a low risk for CRS induction (31, 42). A direct comparison of CAR19 Treg with CAR19 CD8^+^ CTL administration in a xenogeneic GVHD model showed that human CAR19 CD8^+^ CTL–treated mice had significant weight loss, increased clinical scores, and elevated mouse IL-6 levels, in contrast with human CAR19 Tregs (43). In studies described here, we used a syngeneic mouse model of CAR T cell toxicity (36) and found that mice treated with hCAR19 CD8^+^ CTLs had substantial weight loss, increased clinical scores, and 80% mortality, whereas hCAR19 Treg–treated mice had minimal weight loss, low clinical scores, and no mortality. Furthermore, we found that hCAR19 Tregs could be infused on the same day as hCAR19 CD8^+^ CTLs to significantly reduce clinical scores, improve weights, and abrogate lethality. These results align with the function of Tregs to maintain immune homeostasis and dampen excessive, deleterious immune responses (44). These data are relevant as CAR Treg therapies move into the clinic with HLA-A2 CAR Tregs for mismatched kidney transplant recipients (ClinicalTrials.gov NCT04817774).

Tregs use cytolysis as one mechanism to modulate immune responses (26, 45, 46), as well as noncytolytic mechanisms of suppression (22–24). It remains unclear what signals drive Tregs to utilize killing pathways versus other mechanisms of suppression. CAR Tregs have demonstrated minimal killing of antigen-positive cells (17, 43). However, Boroughs et al. in 2019 reported CAR19 human Tregs could kill CD19-expressing cells in vitro via the perforin/GzB pathway (31). Our results align with Boroughs et al., as we found hCAR19 Tregs could kill hCD19 TBL12^luc^ cells in vitro. We found hCAR19 Tregs killed in an antigen-dependent and GzB-independent, perforin-dependent manner. We extend these findings to report that hCAR19 Tregs reduced hCD19 TBL12^luc^ lymphoma cell growth in vivo and enhanced survival when compared with mice treated with tumor cells alone or with tEGFR Tregs. In contrast, Imura et al. in 2020 did not find significant killing in vitro by CAR19 human Tregs, even though their CAR included the CD28 costimulatory domain as did the Boroughs et al. study. Instead, they found CAR19 Tregs suppressed B cell differentiation, proliferation, and antibody production (43). Similar to our allogeneic model, they showed B cells were not pathogenic or necessary for xenogeneic GVHD induction, even though hCD19 B cell expression activated hCAR19 Tregs, thereby facilitating their expansion and immune-suppressive functions (17, 38, 43). With differences in CAR Treg cytolytic potential among CAR Tregs, research is warranted to investigate what factors induce the cytolytic and concurrent immune-suppressive functions in vivo. Nonetheless, cytolytic CAR Tregs may provide a novel therapeutic avenue that tackles key limitations of Treg therapy through suppression of GVHD while substantially reducing the risk for relapse in patients.

Although insufficient Treg purity could confound analysis of Treg cytolytic potential measured, some groups have sorted the top 2% of CD4^+^CD25^+^ T cells (22) or measured in vitro suppressive function immediately prior to adding Tregs to killing assays (30). To address this concern, we sort-purified transduced hCAR19 Tregs from Foxp3-GFP^+^ reporter mice to greater than 95% Foxp3^+^ cells to ensure high Treg purity. Supportive of the suppressive and cytolytic capacity of CAR19 CD4^+^ Tregs, Locafaro and colleagues enforced IL-10 expression in human CD4^+^ T cells to generate a type 1 regulatory–like (Tr1-like) cell that was suppressive and yet acquired cytotoxic activity restricted to myeloid cells, including myeloid malignancies. These human Tr1-like cells suppressed xenogeneic GVHD and potentiated GVT responses (47). Therefore, it is possible that genetic modifications can generate an immune-regulatory and -suppressive cell capable of engaging in cytotoxicity toward a specific cell type.

We chose to evaluate the efficacy of Tregs redirected toward hCD19 on B cells with an FDA-approved construct to facilitate translating our findings to the clinic. The hCAR19 construct used here contains the 4-1BB costimulatory domain associated with activated, effector Tregs (48) and enhanced oxidative phosphorylation, a preferred Treg metabolic program (37). However, recent studies of hCAR19 human Treg studies comparing CD28 and 4-1BB costimulatory domains have found 4-1BB–based CAR Tregs have decreased suppressive function in vitro and in vivo (31, 38), though it can be rescued with transient mTOR inhibition (49). Rapamycin-mediated mTOR inhibition was utilized in our hCAR19 Treg cultures in an effort to maintain Treg purity (50). Interestingly, a study reported that 4-1BB agonist treatment induces a subset of CD4^+^Foxp3^+^ Tregs to eliminate virus-induced tumor cells in mice (51), and the addition of 4-1BB expression in CAR T cells with an intracellular CD28 costimulatory domain improves their proliferation and cytotoxicity (52). The exact role 4-1BB signaling plays in Treg cytolytic potential has not been fully elucidated.

Relapse of the original hematological malignancy following allo-HSCT remains a major challenge and the leading cause of allo-HSCT failure, resulting in a dismal prognosis (53). Salvage chemotherapy, donor lymphocyte infusions (DLIs), and second transplants have low success rates in treatment of relapse (54). Thus, treatment strategies that reduce the risk of relapse and improve treatment outcomes are necessary. Prophylactic DLIs have shown a significant decrease in relapse rates for patients with myeloid leukemias but come at the cost of increased incidence of GVHD (55). Here we show that hCAR19 Tregs given with Tcons suppress aGVHD and tumor growth throughout the duration of our transplants, in stark contrast to mice treated with Tcons alone and Tcons with tEGFR Tregs. This suggests that using cytolytic CAR Tregs specific for antigens of the patient’s hematological condition might significantly reduce both GVHD and relapse. CAR Treg infusion in combination with posttransplant Cy could be particularly effective in preventing GVHD, as the mechanism of posttransplant Cy action appears to depend on Tregs (56). A combination of CAR Treg infusion, posttransplant Cy, and further GVHD prophylaxis with a Treg-supportive drug such as sirolimus (rapamycin) could be a potent anti-GVHD regimen without compromising GVT. Furthermore, cytolytic CAR Tregs may have sufficient antitumor response to be coinfused with DLIs to suppress GVHD while maintaining antitumor effects or be given alone to exploit their dual suppressive and cytolytic properties. Further, cytolytic CAR Tregs offer the possibility of expanding the clinical applications of CAR Treg therapy to autoimmunity, chronic inflammation, and transplantation. CAR Tregs allow targeting of cells responsible for the pathophysiology of certain diseases while simultaneously enhancing Treg expansion and suppression of ongoing inflammatory disease.

Some limitations in our hCD19TG^Tg/0^ model must be considered before extrapolation of hCAR19 Treg efficacy in patients. hCD19TG^Tg/0^ mice that express the hCD19 transgene on B cells have significantly lower B cell frequencies compared with WT mice and elevated B cell hCD19 density when compared with human B cells (36). Although these attributes in B cells represent differences found in our mouse model from B cells in humans, the aggregate effects of high hCD19 density with a relative B cell hypoplasia created a CRS-induced lethality model upon infusion of hCAR19 CD8^+^ T cells. CRS has been reported in immune-deficient mice carrying a high burden of hCD19 lymphoma cells (57), whereas CRS is only observed in syngeneic, immune-competent hCD19TG^Tg/0^ mice that have high hCD19 density coupled with less B cell hypoplasia than hCD19TG^Tg/Tg^ mice (36, 58). Thus, it is possible that the elevated hCD19 density might cause hCAR19 CD8^+^ T cell hyperresponsiveness leading to CRS. Whether hyperresponsiveness is necessary to activate hCAR19 Tregs in our model is not clear. Similarly unclear is the dependency of hCAR19 CD8^+^ CTL persistence on a critical B cell mass or hCD19 density, although in general B cell aplasia has been ascribed to be a surrogate for persistent CAR19 CD8^+^ CTLs. Future studies may consider boosting hCD19 engagement by periodic infusions of hCD19-Fc protein. Regardless of the mechanistic underpinnings of the CRS, we have demonstrated a potentially novel mechanism by which hCAR19 Tregs can be activated in vivo to suppress GVHD and maintain GVT responses without the risk of CRS.

In summary, to our knowledge, this is the first report that a CAR Treg therapy can control tumor growth in vivo with the capacity to suppress GVHD without CAR-associated toxicities. These findings expand our understanding of CAR Treg function and inform future Treg therapy design and application. The potentially novel approach of redirecting CAR Tregs to tumor antigens is a therapeutic avenue with the potential to improve outcomes in allo-HSCT patients by reducing the risk of GVHD and relapse.

## Methods

### Mice.

BALB/c, C57BL/6, and CD45.1 female mice were purchased from Charles River Laboratories. C57BL/6 perforin 1–KO mice were purchased from The Jackson Laboratory. Thomas Tedder (Duke University School of Medicine, Durham, North Carolina, USA) provided hCD19TG^Tg/Tg^ mice. We bred hCD19TG^Tg/Tg^ mice with C57BL/6 mice to generate hCD19TG^Tg/0^. We also bred hCD19TG^Tg/Tg^ mice with BALB/c mice to generate hCD19^Tg/0^ CB6F1. B6 Foxp3-GFP knock-in mice were kindly provided by Vijay Kuchroo (Harvard University, Boston, Massachusetts, USA) and bred in our animal colony. B6 Foxp3-GFP-Luciferase mice were bred in our animal colony. All mice were housed in a specific pathogen–free facility and used with University of Minnesota Institutional Animal Care and Use Committee approval.

### Cell culture.

Tregs and T cells were cultured in Expansion Media, DMEM-based media (DMEM, high glucose, pyruvate; Thermo Fisher Scientific) supplemented with 10% fetal bovine serum (FBS; Premium; Atlanta Biologicals), 10 mM HEPES (MilliporeSigma), 1× nonessential amino acids (NEAA; Thermo Fisher Scientific), 1× penicillin/streptomycin (MilliporeSigma), 50 μg/mL gentamicin (gentamicin sulfate, liquid, Corning), and 55 mM 2-mercaptoethanol (MilliporeSigma). TBL12 and hCD19 TBL12^luc^ cell lines were cultured in RPMI-1640 (Thermo Fisher Scientific) supplemented with 10% FBS (Thermo Fisher Scientific), 10 mM HEPES (MilliporeSigma), 1× penicillin/streptomycin (MilliporeSigma), 1× NEAA (Fischer Scientific), and 55 mM 2-mercaptoethanol (MilliporeSigma).

### Isolation of primary murine Tcons and Tregs.

CD4^+^ and CD8^+^ T cells (Tcons) were purified from spleens by negative selection using biotin-labeled anti-CD19 (1D3), -B220 (RA3-6B2), -CD11b (M1/70), -CD11c (N418), -NK1.1 (PK136), -CD49b (DX5) -CD25 (PC61.5), -γδ, (GL3), and –TER-119 (TER-119) (StemCell Technologies), followed by streptavidin RapidSphere depletion with EasySep magnet (StemCell Technologies). Tregs were purified from lymph nodes and spleens using negative selection as above but with the addition of anti-CD8 (53-6.7, StemCell Technologies) and in the absence of anti-CD25 to enrich for CD4^+^CD25^+^ T cells. CD4^+^CD25^+^ T cells were then incubated with PE-labeled anti-CD25 antibody (PC61.5, eBioscience), followed by anti-PE beads (Miltenyi Biotec), and CD25^+^ cells were selected via magnetic columns (Miltenyi Biotec). Tregs from WT mice were stained with anti-CD4 (BioLegend; GK1.5), anti-CD25 (Thermo Fisher Scientific; PC61.5), and Fixable viability dye eFluor 780 (eBioscience) to be sorted as CD4^+^CD25^hi^ cells. Treg from Foxp3-GFP mice were sorted as CD25^hi^GFP^+^ from CD4^+^ T cells using a BD LSRII/Fortessa/Canto.

### Plasmid construction and retroviral transduction.

The hCAR19 construct was subcloned from a lentivirus into an MT71 retroviral vector, which was optimized for T cell expression. Tregs were activated with anti-CD3/CD28 Dynabeads (Thermo Fisher Scientific) at a 3:1 bead-to-Treg ratio in Expansion Media with 2,000 IU/mL of recombinant human IL-2 (Proleukin) and 5 nM rapamycin (MilliporeSigma). On day 3 after Treg harvest, a 48-well plate was coated with RetroNectin (Takara); retrovirus containing the tEGFR or hCAR19-tEGFR construct was added and spinoculated for 2 hours at 2,000 rpm. Supernatant was discarded and 1 × 10^6^ Tregs were added per well and spinoculated for 15 minutes at 1,500 rpm. After spinoculation, Tregs were incubated at 37°C, 5% CO_2_, with a media change every 48 hours. Tcons were activated with anti-CD3/CD28 Dynabeads at a 1:1 Tcon/bead ratio in Expansion Media with 100 IU/mL of recombinant human IL-2. On day 2 after Tcon harvest, a 24-well plate was coated with RetroNectin; retrovirus containing the tEGFR or hCAR19-tEGFR construct was added and spinoculated for 2 hours at 2,000 rpm. Supernatant was discarded and 0.5 × 10^6^ Tcons were added per well and spinoculated for 15 minutes at 1,500 rpm. After spinoculation, Tcons were incubated at 37°C, 5% CO_2_, with a media change every 48 hours. On day 8 after harvest, we measured transduction efficiency via tEGFR expression. To enrich for tEGFR^+^ transduced cells, Tregs or Tcons were stained with anti-human EGFR (AY13) and then incubated with anti-PE beads (Miltenyi Biotec) to be selected via magnetic column.

### aGVHD and GVT model.

hCD19TG^Tg/0^ recipients were lethally irradiated with 11 Gy a day prior to transplantation, then injected IV with 5 × 10^6^ BALB/c BM; BM with 2.5 × 10^6^ CD25^–^ BALB/c Tcons; or BM with Tcons and 1.25 × 10^6^ hCAR19 Tregs or CTLs, tEGFR Tregs, or CTLs. Mice were evaluated for daily survival, weights, and evidence of clinical GVHD as previously described (59). hCD19 TBL12^luc^ is a B cell lymphoma cell line of B6 origin (60) that has been modified to express human CD19 antigen on B cells, as well as to express luciferase. hCD19TG^Tg/0^ were lethally irradiated with 11 Gy a day prior to transplantation. hCD19TG^Tg/0^ were injected IV with 5 × 10^6^ TCD BM from BALB/c mice alone or injected with TCD BM plus 2.5 × 10^6^ CD25^–^ Tcons and 10^4^ hCD19 TBL12^luc^, or with TCD BM with Tcons and hCD19 TBL12^luc^ and 1.25 × 10^6^ of either hCAR19 or tEGFR Tregs. Tumor growth was monitored by luciferase imaging of lymphoma cells. To directly measure Treg antitumor responses in vivo, we used a similar experimental design, except we set up a syngeneic transplant where each group received 5 × 10^6^ C57BL/6 TCD BM with 1.0 × 10^6^ hCAR19 or tEGFR Tregs in the absence of Tcons.

### BLI studies.

We intraperitoneally injected firefly luciferin substrate (0.1 mL at 30 mg/mL, Promega) into recipient mice and waited 5 minutes prior to imaging. The Xenogen IVIS imaging system was used, and data were analyzed using the Living Image 3.0 Software.

### In vitro Treg suppression assay.

TCD splenocytes and B cells were isolated from either WT C57BL/6 mice or hCD19TG^Tg/0^ and mixed at 1:1 ratio to generate APCs. CD25-depleted T cells were isolated from BALB/c mice and stained with 2.5 μM CTV (Life Technologies) for 10 minutes at 37°C to track proliferation. Tregs were also isolated from BALB/c mice and transduced as described above. Anti-CD3 (0.25 μg/mL, clone 145-2C11, eBioscience) was added to the assay to stimulate T cell proliferation.

### Colon lymphocyte isolation.

We isolated the lamina propria lymphocytes using a protocol previously described (61). Briefly, mice were sacrificed on day 14 after allo-HSCT; the colons were harvested and flushed with PBS containing 10% FBS. Each colon was cut into pieces and washed twice for 10 minutes at 37°C with a cell dissociation buffer (Ca/Mg-free PBS with 5 nM EDTA and 10 nM HEPES). Tissues were washed once with PBS containing 10% FBS for 5 minutes at 37°C, then cut into smaller pieces prior to being treated 3 times for 20 minutes at 37°C with a digestion buffer: 1 mg/mL collagenase D (Roche), 0.15 IU/mL Dispase (Worthington), and 0.5 mg/mL DNase I (Roche) in Ca/Mg-free PBS containing 10% FBS. Lymphocytes were purified using a 40% and 80% Percoll (MilliporeSigma) gradient.

### Cytotoxicity assays.

For the IncuCyte killing assay, we used TBL12 or hCD19 TBL12^luc^ cells, which were labeled with CellTrace Far Red (Thermo Fisher Scientific) and incubated with either hCAR19 or tEGFR Tregs at a 2:1 E/T ratio for 48 hours. IncuCyte Caspase-3/7 green apoptosis assay reagent (Essen Biosciences) was also added per well. Images were taken every few minutes, and the number of apoptotic cells per well was quantified using the IncuCyte Caspase-3/7 green apoptosis assay reagent and the IncuCyte Zoom platform (Essen Biosciences). For the flow cytometry killing assay, we used hCD19 TBL12^luc^ cells, which were stained using the CellTrace Far Red (Thermo Fisher Scientific) and incubated with either hCAR19 or tEGFR Tregs, CD4^+^ T cells, or CD8^+^ T cells at a 5:1 E/T ratio for 48 hours. Killing was calculated through viability measured using the Fixable Viability Dye of the Far Red–stained tumor cells (62).

### In vivo toxicity measurement.

We used a previously published model where effectors are adoptively transferred into hCD19TG^Tg/0^ recipient mice a day after receiving a lympho-depleting dose of 300 mg/kg of Cy (Cytoxan) (36). A total of 3 × 10^6^ effectors were injected IV into the tail vein. Survival, weights, and clinical scores were recorded daily. Scores were assigned as 0–2 on each of the 4 criteria: activity, fur texture, posture, and weight. A score of 0 compared with 8 indicates a healthy and moribund mouse, respectively (59).

### Metabolic flux analysis.

With XFe-96 Extracellular Flux Analyzer (Seahorse Bioscience), we measured oxygen consumption rates (OCRs) and extracellular acidification rates (ECARs) using the XF media (modified DMEM containing 2.5 mM glucose, 2 mM glutamine, and 1 mM sodium pyruvate). OCR was measured in response to 1 μM oligomycin, 1 μM fluorocarbonyl cyanide phenylhydrazone, and 1 μM antimycin, while ECAR was measured in response to 20 mM glucose, 1 μM oligomycin, and 80 mM 2-deoxyglucose. Prior to Seahorse analysis Tregs were stimulated in an hCD19 Fc–coated plate (R&D Systems, 0.02 μg/μL).

### Flow cytometry.

Fluorochrome-conjugated monoclonal/polyclonal antibodies used in our studies were anti-mouse CD4 (GK1.5, BD Biosciences), anti-mouse CD25 (PC61.5l, eBioscience), anti-mouse Foxp3 (FJK-16s, eBioscience), anti-mouse CD8 (53-6.7, eBioscience), anti-human CD19 (H1B19, eBioscience), anti-human EGFR (AY13, BioLegend), anti-mouse CD45.1 (A20, eBioscience), anti-mouse CTLA-4 (UC10-4B9, eBioscience), anti-mouse neuropilin 1 (eDS304M, eBioscience), anti-mouse Lag-3 (C9B7W, eBioscience), anti-mouse CD11c (N418, eBioscience), anti-mouse IFN-γ (XMG1.2, eBioscience), anti-mouse TNF-α (MP6-XT22, eBioscience), anti-mouse Fas (SA367H8, BioLegend), anti-mouse FasL (MFL3, eBioscience), anti-mouse perforin (eBioMAK-D, eBioscience), anti-mouse GzB (NGZB, eBioscience), anti-mouse GzA (3G8.5, BioLegend), anti-mouse CD107α (1D4B, eBioscience), anti-mouse CD71 (R17217, eBioscience), anti-mouse CPT1a (8F6AE9, Abcam), anti-mouse Glut1 (ER3915, Abcam), and Fixable viability dye eFluor 780 (BD Biosciences). Intracellular staining was performed using the fixation/permeabilization concentrate (catalog 5123-43) and diluent (catalog 5223-56) buffer solutions and IC buffer (catalog 8333-56), according to the eBioscience Foxp3 staining kit. Stained cells were analyzed on an LSR Fortessa flow cytometer (BD Biosciences), and data were analyzed using FlowJo software (Tree Star).

### Statistics.

Data are presented as mean ± SD. Statistical analyses were performed using Student’s unpaired 1-tailed *t* test with Bonferroni correction for multiple comparisons when necessary or a log rank (Mantel-Cox) test in survival studies using GraphPad Prism version 9 software. *P* values less than 0.05 were considered statistically significant.

### Study approval.

Animal studies were conducted in accordance with a protocol reviewed and approved by the IACUC of the University of Minnesota (2103A38904).

## Author contributions

SBW designed and performed experiments, analyzed results, and wrote the manuscript. MLL designed and performed experiments and analyzed results. SJ, CSMH, JHL, MH, GT, and EAA performed experiments, discussed results, and edited the manuscript. MCJ and JEW edited the manuscript. MJO generated retroviral constructs and edited the manuscript. GT, BHK, AS, CAP, and BRB contributed to experimental design, discussed results, and edited the manuscript. For SBW and ML’s author assignments, ML started the project but then moved back to France, and a large effort was made by SBW, who provided much of the data for the paper.

## Supplementary Material

Supplemental data

## Version 1 08/02/2022

In-Press Preview

## Version 2 09/08/2022

Electronic publication

## Version 3 09/14/2022

Ethan G. Aguilar's name was corrected
