# Supplementary material for: Murine CAR19 Tregs suppress acute graft-versus-host disease and maintain graft-versus-tumor responses
Source: JCI Insight. 2022 Sep 8;7(17):e160674. doi: 10.1172/jci.insight.160674 (PMC9536261; doi:10.1172/jci.insight.160674)
Supplement: Supplemental data [file jciinsight-7-160674-s240.pdf]

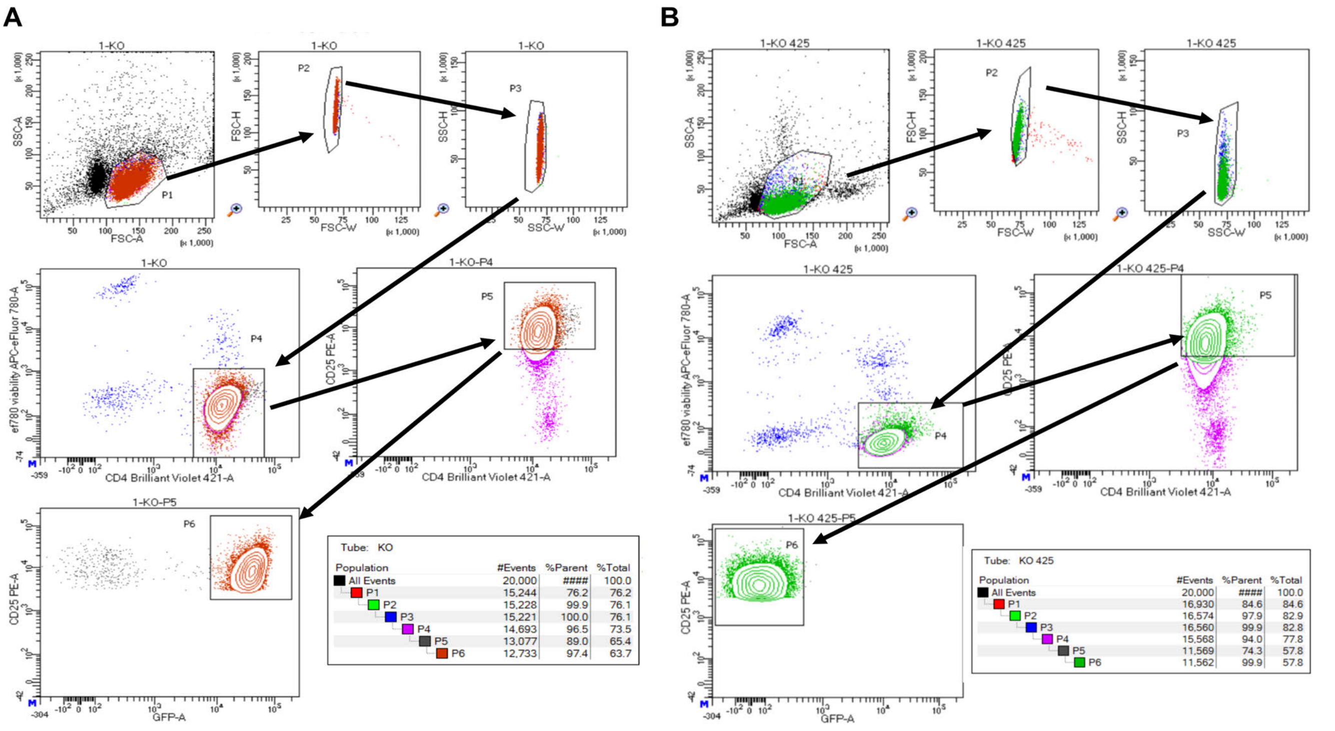

Supplementary Figure 1. Gating strategy for Treg sorting. (**A**) Sorting $CD25^{+}GFP^{+}$ Treg from Foxp3 GFP^{+} knock in (KI) transgenic mice. (**B**) Sorting $CD25^{hi}$ Treg from wildtype mice.

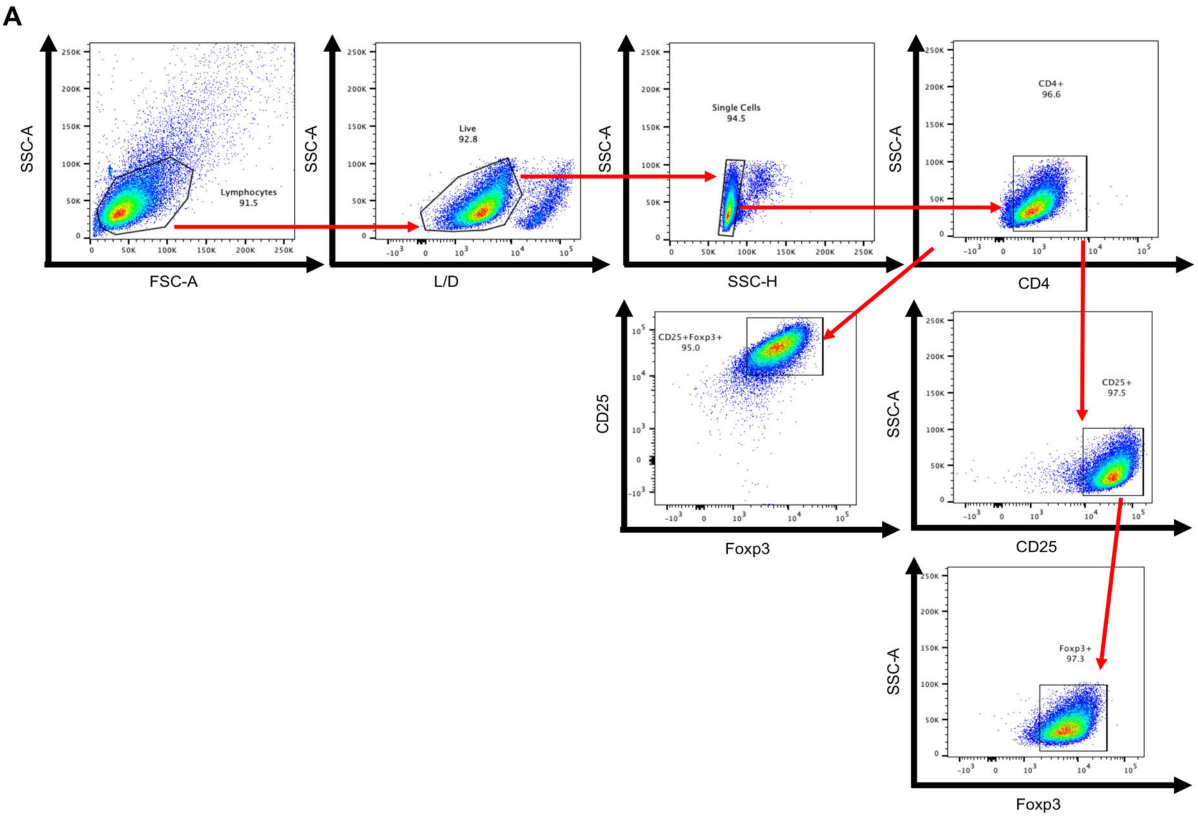

Supplementary Figure 2. Gating strategy for Treg purity assessment. (**A**) Representative flow cytometry plots of each population gated to assess Treg purity prior to experimental use. L/D, live/dead.

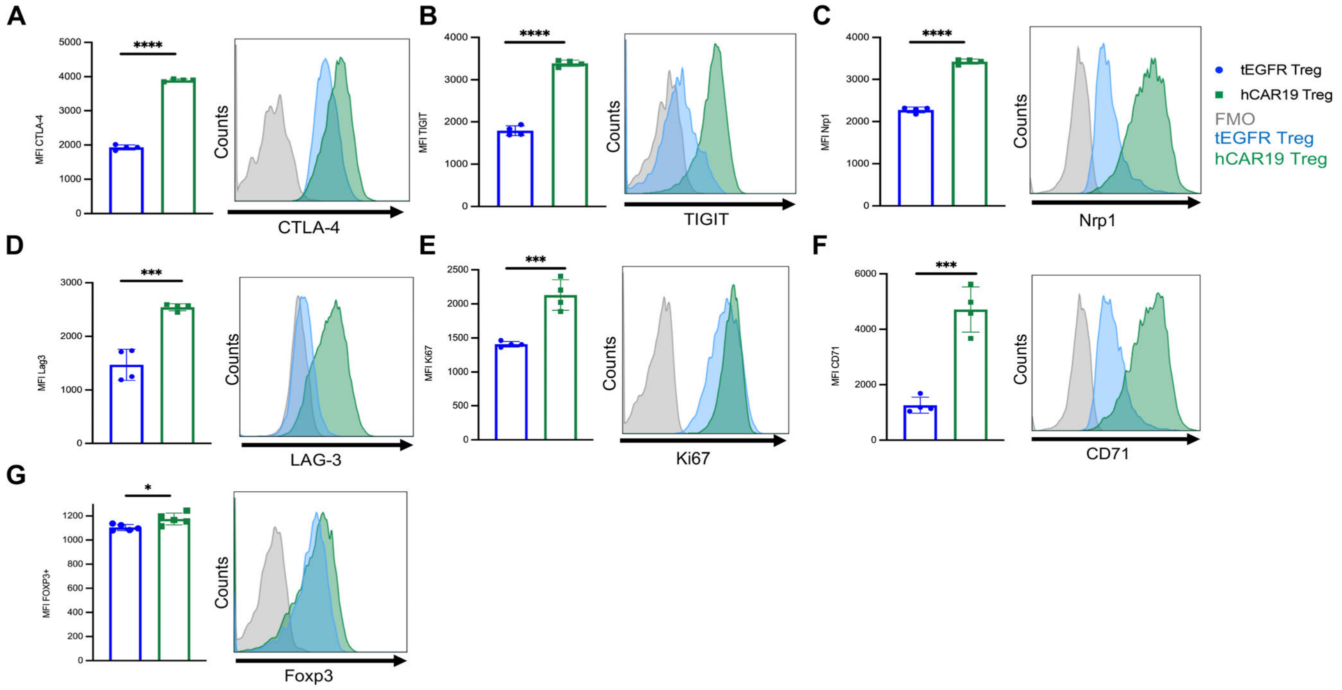

Supplementary Figure 3. Antigen specific activation increases hCAR19 Treg expression of suppression, proliferation and activation markers compared to tEGFR Treg. (**A-G**) MFI of CTLA-4, TIGIT, Nrp1, LAG-3, Ki67, and Foxp3 after 48 hour stimulation of Treg in a hCD19 Fc coated plate. hCAR19, n=4; tEGFR, n=4. Data is representative from two independent experiments. FMO, fluorescence minus one. Student t test was used for statistical analysis. Error bars indicate the standard deviation of the mean. ns: no significance; *:<0.5 ; **: <0.01; ***:<0.001; ****:<0.0001.

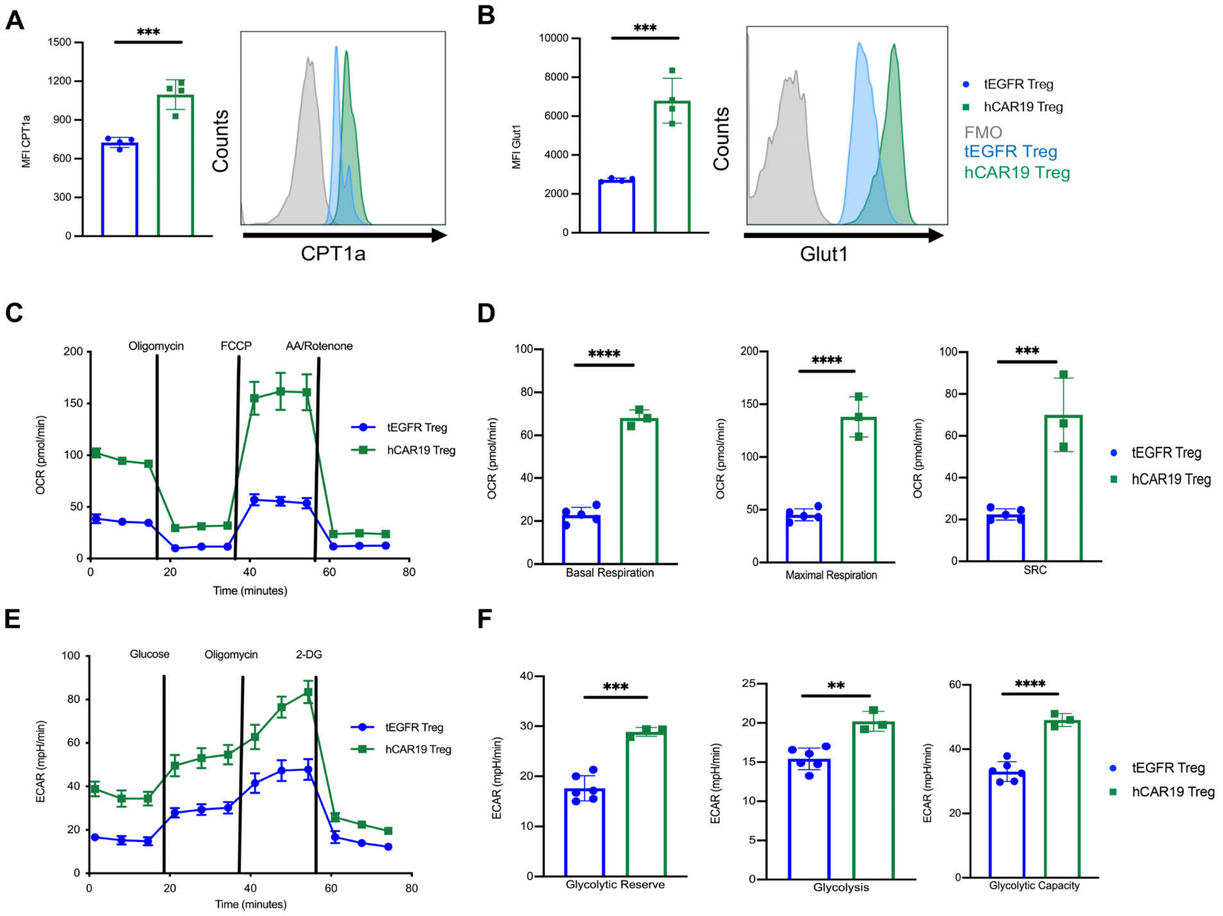

Supplementary Figure 4. Antigen specific activation increases hCAR19 Treg metabolic fitness compared to tEGFR Treg. hCAR19 and tEGFR Treg were stimulated 48 hours in a hCD19 Fc coated plate. (**A**-**B**) Mean fluorescence intensity (MFI) of metabolic markers CPT1a and Glut1. hCAR19 Treg, n=4; tEGFR Treg, n=4. FMO, fluorescence minus one. (**C**) Oxygen consumption rate (OCR) analysis. (**D**) Quantification of basal respiration, maximal respiration, and spare respiratory capacity (SRC). hCAR19 Treg, n=3. tEGFR Treg, n=5. (**E**) Extracellular acidification rate (ECAR). (**F**) Quantification of glycolytic reserve, glycolysis, and glycolytic capacity. hCAR19 Treg, n=3; tEGFR Treg, n=6. Data is representative from two independent experiments. Student t test was used for statistical analysis. Error bars indicate the standard deviation of the mean. ns: no significance; *:<0.5 ; **: <0.01; ***:<0.001; ****:<0.0001.

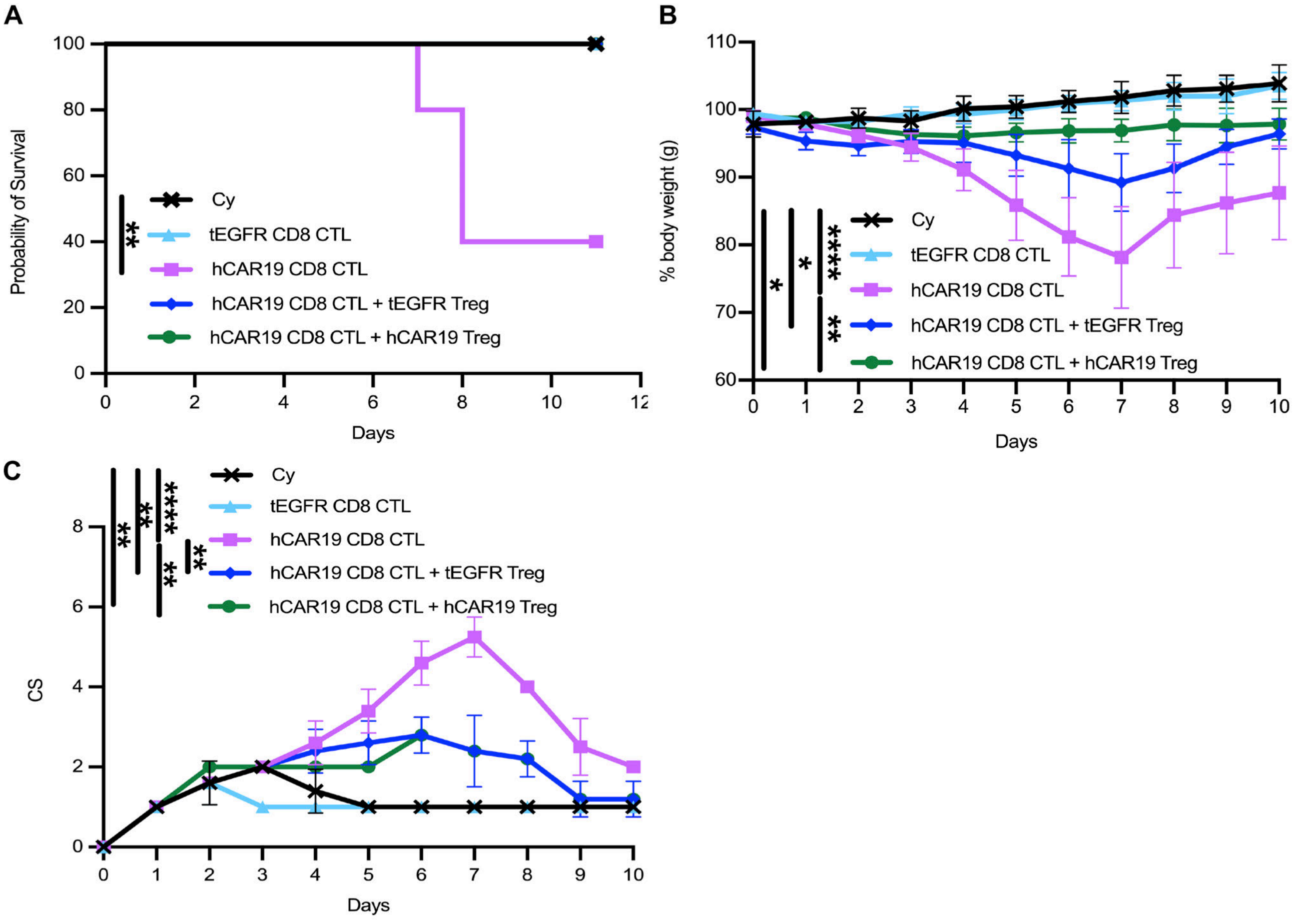

Supplementary Figure 5. hCAR19 Tregs suppress system toxicities induced by hCAR19 CD8 CTL in a syngeneic mouse model. $hCD19TG^{Tg/0}$ recipient mice injected with 300 mg/kg of cyclophosphamide (Cy) a day prior to adoptive cell transfer (ACT) with $3x10^6$ C57BL/6 hCAR19 or tEGFR CD8 T-cells (CTL); some hCAR19 CD8 CTL groups also received $1.5x10^6$ of either tEGFR Tregs or hCD19 Tregs. (**A**) Survival. (**B**) Percent body weight. (**C**) Clinical scores. (**A**-**C**) Cy, n=5; tEGFR CD8 CTL, n=5; hCAR19 CD8 CTL, n=5**;** hCAR19 CD8 CTL + tEGFR Treg, n=5; hCAR19 CD8 CTL + hCAR19 Treg, n=5. Statistics are shown from day 7 after ACT. Student t test with correction for multiple comparison was used for statistical analysis. Log rank test was used to analyze survival curves. Error bars indicate the standard deviation of the mean. ns: no significance; *:<0.5 ; **: <0.01; ***:<0.001; ****:<0.0001.

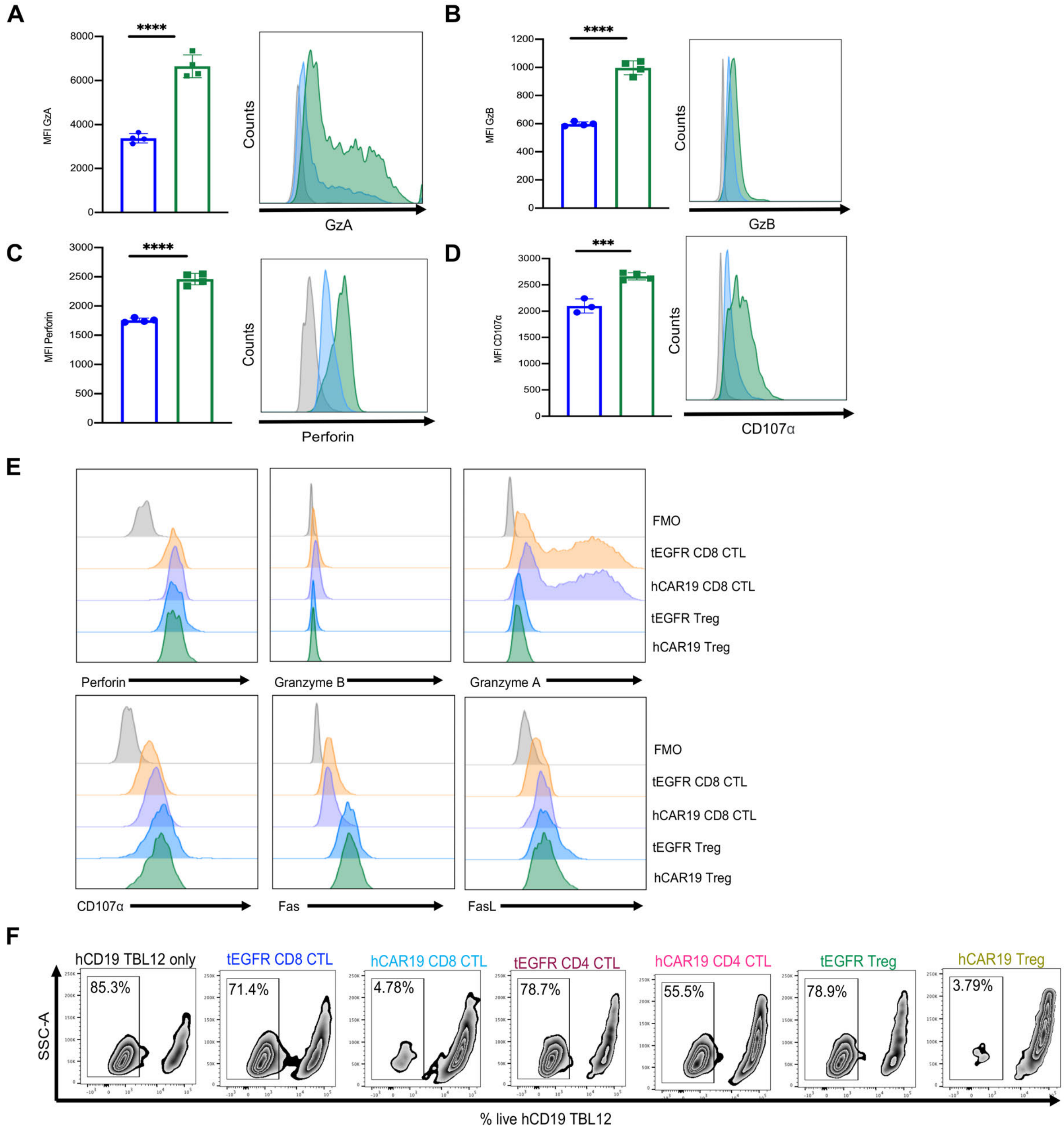

Supplementary Figure 6. hCAR19 Treg have increased expression of killing markers after antigen specific activation and engage in in vitro killing of hCD19 TBL12. (**A**-**D**) MFI of granzyme A (GzA), granzyme B (GzB), perforin (perf), and CD107α after 48 hour stimulation in a hCD19 Fc coated plate. hCAR19 Treg, n=4; tEGFR Treg, n=4.(**E**) Representative histogram plots of perforin, CD107α , GzA, GzB, FasL and Fas prior to hCD19 stimulation. (**F**) Representative flow plots of a 48 hour flow cytometry in vitro killing assay using hCD19 TBL12^{luc} with sorted Foxp3^{+}GFP^{+} Treg the day of experiment from Figure 4H. Student t test was used for statistical analysis. Error bars indicate the standard deviation of the mean. ns: no significance; *:<0.5 ; **: <0.01; ***:<0.001; ****:<0.0001.

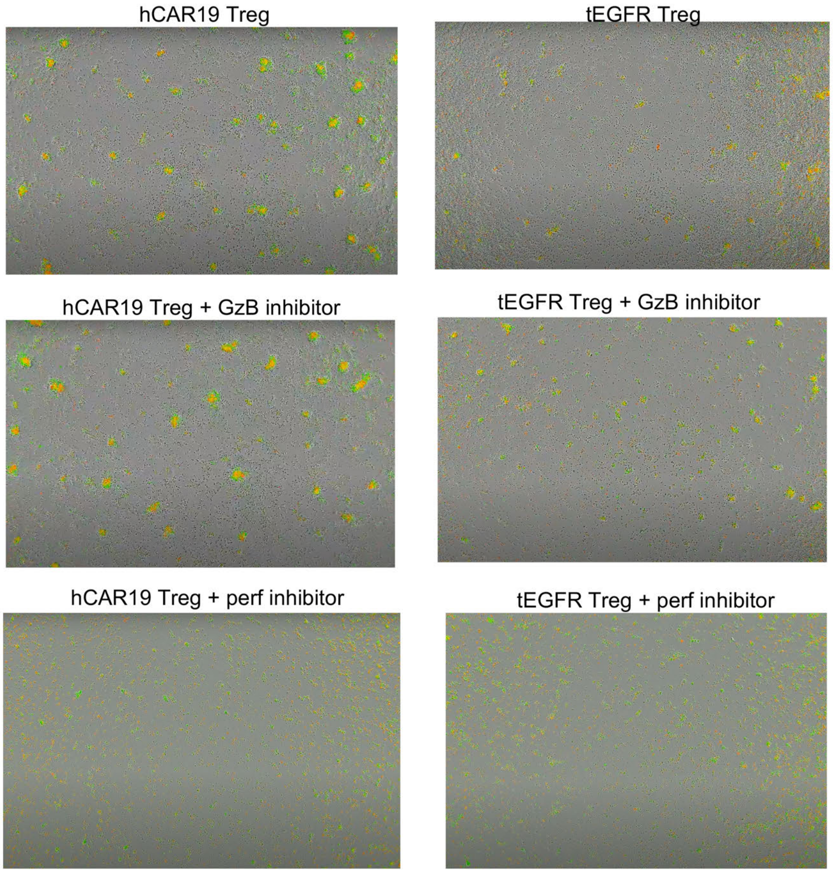

Supplementary Figure 7. hCAR19 Tregs, unlike tEGFR Treg, engage in in vitro killing of hCD19 TBL12 in a perforin dependent, GzB independent manner. Representative videos of IncuCyte in vitro killing assay in Figure 4I using the perforin (per) inhibitor concanamycin A (CMA) and granzyme B (GzB) inhibitor Z-AAD-CMK with hCD19 TBL12^{luc} tumor. E:T ratio used was 2:1. n=5 for all groups, except for hCAR19 Treg and hCAR19 Treg GzB had n=3.

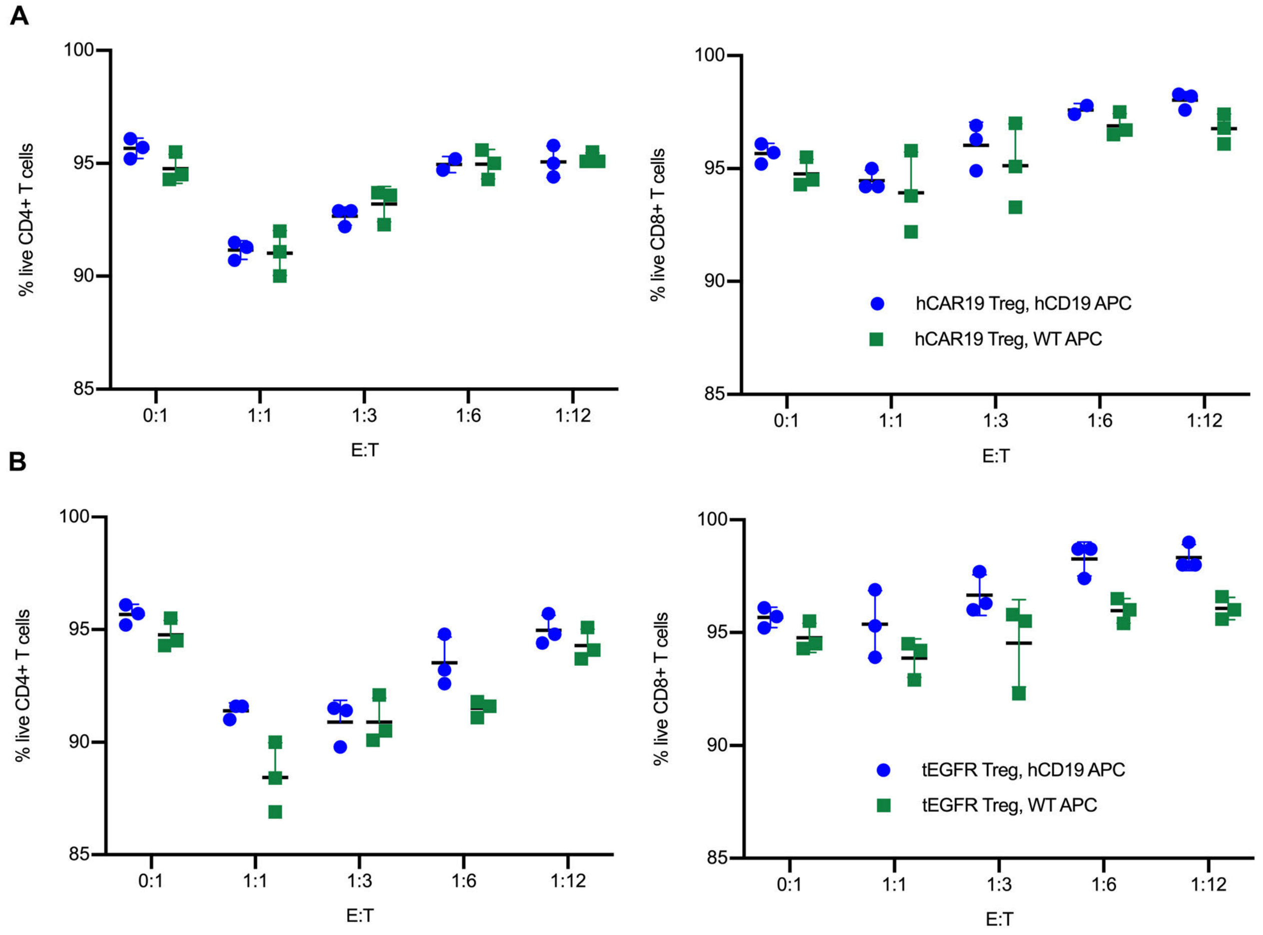

Supplementary Figure 8. hCAR19 Treg do not engage in in vitro killing of Tcon when in the presence of hCD19 B-cells. (**A**) Frequency of live CD4 and CD8 T-cells cocultured for 72 hours with hCAR19 Treg in the presence of either hCD19 APC or WT APC. (**B**) Frequency of live CD4 and CD8 T cells co-cultured for 72 hours with tEGFR Treg in the presence of either hCD19 APCs or WT APCs. n=3 for all groups. Data is representative of two independent experiments.

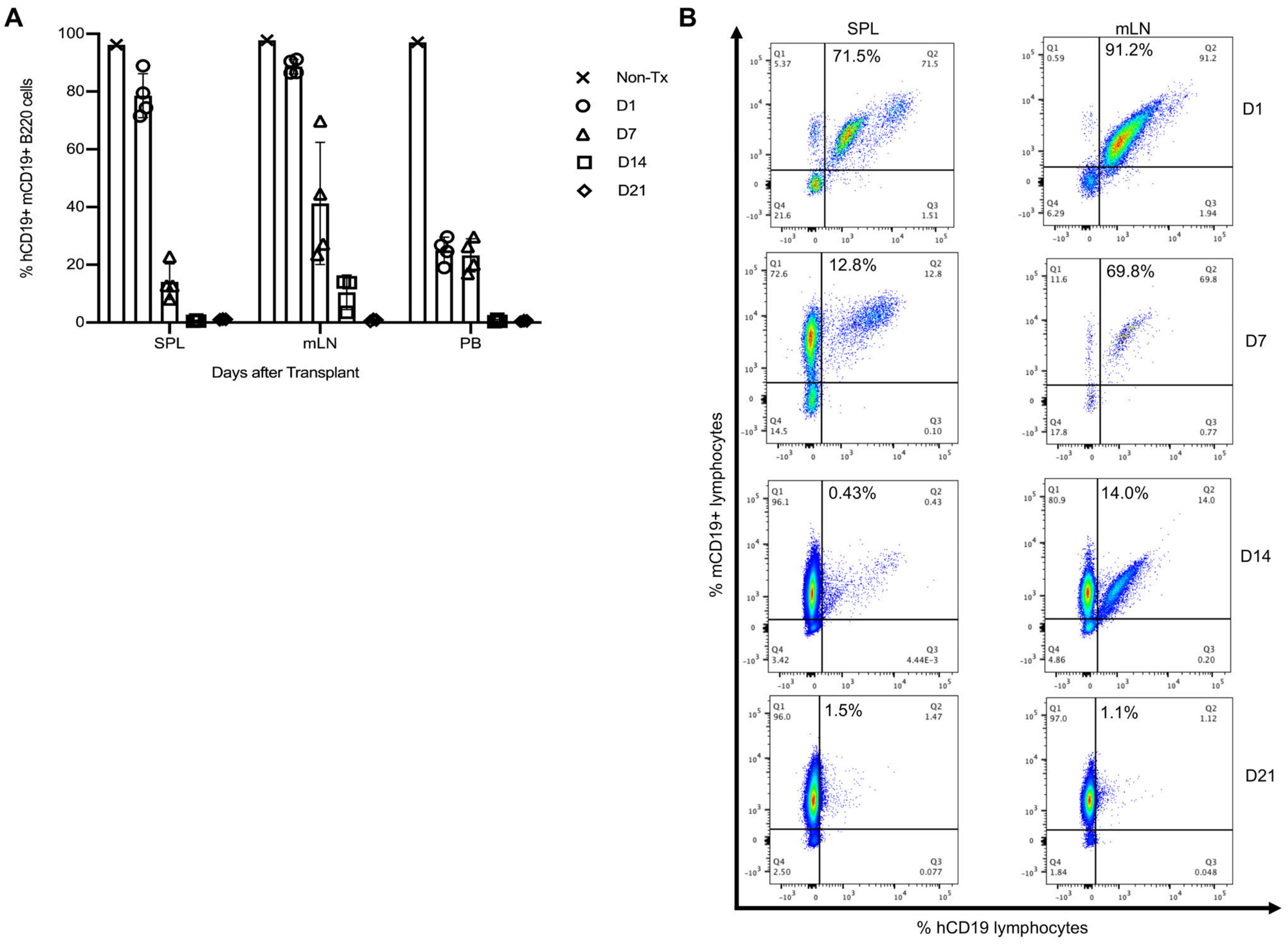

Supplementary Figure 9. hCD19 B-cells are present in recipient hCD19TG$^{TG/0}$ mice after allo-HSCT. (**A**) Percent double positive hCD19, mCD19 B220 lymphocytes in the SPL, mLN, and PB in hCD19TG$^{TG/0}$ recipient mice following a lethal irradiation prior to transplantation with BALB/c bone marrow. Non-tx, non-transplanted mouse; SPL, spleen; mLN, mesenteric lymph node; PB, peripheral blood; allo-HSCT, allogeneic hematopoietic stem cell transplantation. (**B**) Representative flow cytometry plots of hCD19 and mCD19 populations in the SPL and mLN on day 1, 7, 14, and 21 after transplant.

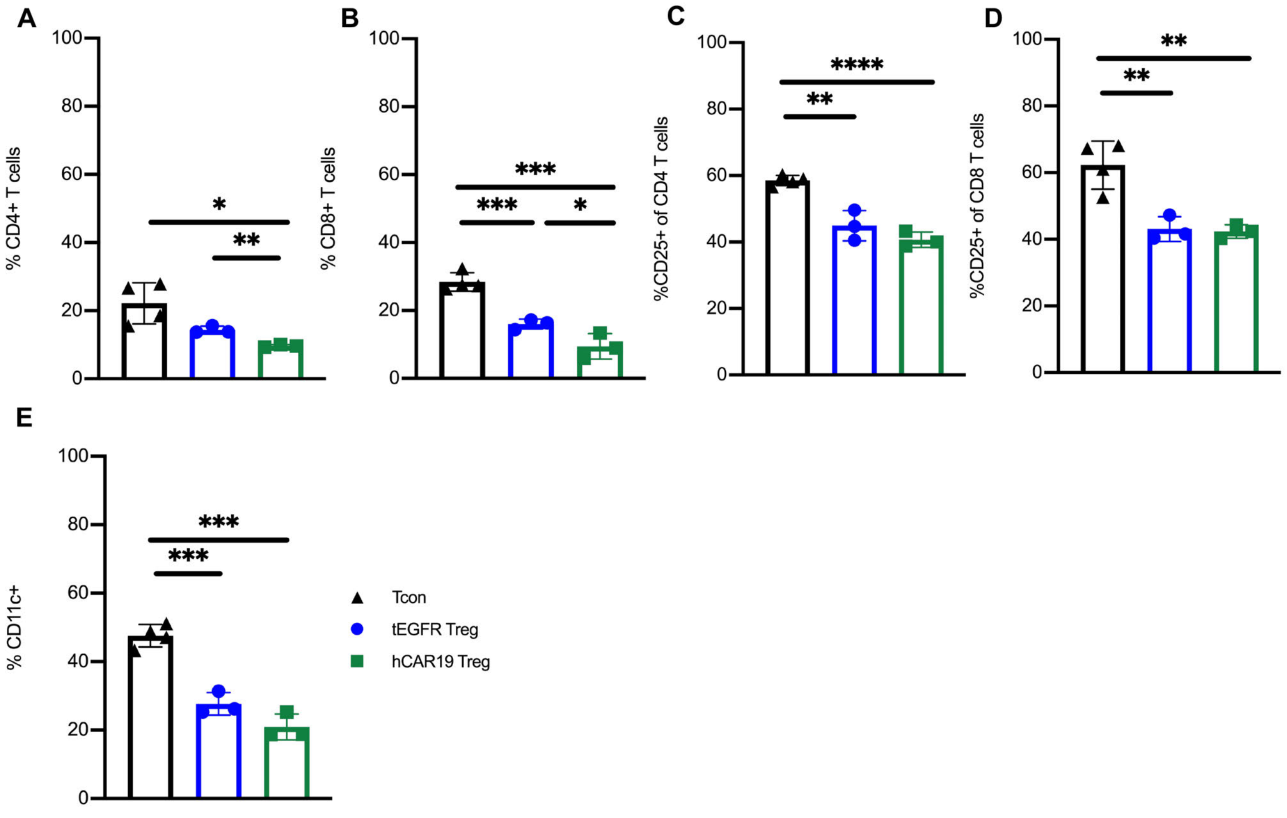

Supplementary Figure 10. hCAR19 Treg suppress Tcon and CD11c monocytes and DC on day 5 after allo-HSCT. Frequency of CD4 and CD8 T-cells (**A**-**B**), CD25⁺ CD4 and CD8 T cells (**C**-**D**), and CD11c lymphocytes (**E**) in the spleen of hCD19TG$^{TG/0}$ recipient mice on day 5 after undergoing a lethal irradiation prior to receiving BALB/c bone marrow with Tcon and either hCAR19 or tEGFR Treg. Data is representative from two intendent experiments. Student t test with correction for multiple comparison was used for statistical analysis. Error bars indicate the standard deviation of the mean. ns: no significance; *:<0.5 ; **: <0.01; ***:<0.001; ****:<0.0001.
